# Improved imaging interfaces on a co-registered ultrasound and optical microscopy multiscale system

**DOI:** 10.1117/1.JBO.31.1.016002

**Published:** 2025-12-25

**Authors:** Jonathan Hale, Nathan Cudworth, Renata Farrell, Aarushi Bhargava, Kevin W. Eliceiri, Ivan Rosado-Mendez

**Affiliations:** a University of Wisconsin–Madison, School of Medicine and Public Health, Department of Medical Physics, Madison, Wisconsin, United States; b University of Wisconsin–Madison, College of Engineering, Department of Biomedical Engineering, Madison, Wisconsin, United States; c University of Wisconsin–Madison, School of Medicine and Public Health, Department of Radiology, Madison, Wisconsin, United States

**Keywords:** nonlinear optics, second-harmonic generation, multiscale, multimodal, ultrasound, acoustic clutter, collagen

## Abstract

**Significance:**

Integrating ultrasound (US) with multiphoton microscopy (MPM) enables comprehensive tissue characterization by combining contrast mechanisms across spatial scales. However, prior implementations suffer from degraded US image quality at the glass optical window arising from off-axis acoustic noise (clutter).

**Aim:**

We aimed to reduce clutter near the optical window without compromising the MPM image quality using different optical window materials combined with different ultrasound beamforming techniques.

**Approach:**

Clutter in B-mode images was measured for glass and several optically clear polymer films using physically or synthetically focused US beamforming with varied focal configurations (f/#) and with acoustic coupling beneath the window. MPM image quality with the material optical windows was assessed via second harmonic generation (SHG) point spread function (PSF) measurements. We evaluated SHG-based automated collagen fiber alignment quantification with the optical windows in rat tail tendon samples and collagen gels.

**Results:**

Candidate materials included polymethyl pentene (PMP), cyclin olefin copolymer (COC), polycarbonate (PC), polyethylene terephthalate, polymethyl methacrylate, and an Ibidi^®^ polymer coverslip. At receive f/#≥2, COC and Ibidi^®^ reduced acoustic clutter by >40% compared with glass with physical focusing regardless of physically or synthetically focused beams or acoustic coupling. Collagen gel imaged with COC and high f/# showed clearer ultrasound speckle close to the optical windows. COC, Ibidi^®^, PMP, and PC showed minimal SHG PSF differences from glass and collagen alignment errors <5% relative to glass.

**Conclusions:**

COC or Ibidi^®^ optical windows significantly improve US image quality without compromising MPM quality. These enhancements support future multimodal studies with high-quality, co-registered US and MPM data.

## 1 Introduction

The combination of tissue imaging at different spatial scales can unveil the mechanisms by which macroscopic changes (several millimeters to centimeters) result from tissue remodeling at the nano- or micro-scales. Multimodal imaging can also overcome the limitations of individual modalities by building on the strengths of each other. For example, ultrasound imaging at diagnostic frequencies (1 to 30 MHz) can overcome the limiting penetration of optical imaging, whereas the latter allows for visualization of micro-scale structures that ultrasound cannot resolve. Moreover, a direct correlative multimodal imaging device that combines the hardware of multiple imaging modalities would allow for streamlined sequential multimodal acquisition without moving the imaging subject.^1^

Our group has introduced a direct correlative multimodal imaging system that combines multiphoton microscopy (MPM) and high-frequency ultrasound (US, >10  MHz).^2^^–^^4^ On this system, tissue samples can be translated on a motion stage to allow for three-dimensional (3D) correlated data acquisition for both MPM and US. Multiphoton microscopy probes tissue with a single wavelength of light and uses optical filters to detect light (typically backscattered light) at other wavelengths. Multiphoton microscopy can image extrinsic fluorescence^5^ (use of fluorescent dyes, proteins, or other contrast agents added to the sample) and intrinsic fluorescence^6^ (naturally occurring fluorescence). US probes tissue with broadband ultrasound pulses and reconstructs an image from the scattered echoes produced by changes in acoustic impedance (the product of acoustic sound speed and material density).^7^ The analysis of the phase and magnitude of the ultrasound echoes leads to a wide range of imaging modes that inform on microstructural tissue features, blood flow, and stiffness, among other sources of contrast.^8^ Of particular interest is a set of US techniques referred to as backscatter quantitative ultrasound (bQUS) which aim at providing quantitative information related to tissue microstructure. This information is obtained from the statistical and spectral (frequency-dependent) analysis of backscattered echoes produced by sub-millimetric acoustic scattering sources. This multimodal scope is well-suited to create spatially co-registered MPM/US images that could help explain the correlation between large-scale (hundreds of microns) US structures or quantitative bQUS features and micro-scale MPM tissue features. Moreover, the tissue sample can be sequentially imaged with MPM and US without the need to move the sample, avoiding the risk of tissue deformation or damage from handling.^3^

Correlated US and second harmonic generation (SHG) imaging can be done indirectly. For example, Reusch et al.^9^ performed bQUS assessment and SHG microscopy of human cervix specimens from hysterectomy samples. The SHG images and bQUS features were registered through edge detection algorithms performed on the SHG images. It was found that bQUS data suggesting aligned collagen fibers was corroborated by the alignment estimates derived from curvelet analysis of the registered SHG images. The preliminary data from this study suggested that aligned collagen fiber microstructure can be detected acoustically.^9^ Another study by Peralta et al.^10^ performed ultrasound shear wave elastography (SWE, an ultrasound-based technique used to quantify stiffness of tissue) of the cervix of pregnant ewes and then imaged the same regions with SHG microscopy. This study found that the collagen fiber structures found by SHG could be closely related to the stiffness measurements made from SWE, though the two modalities were not registered. The multimodal imaging approaches used in these studies were limited by a lack of integration of the different imaging modalities, making it difficult to register the ultrasound and microscopy images, especially if tissue sample deformation occurred in the time among imaging modalities.^1^

**Fig. 1:**
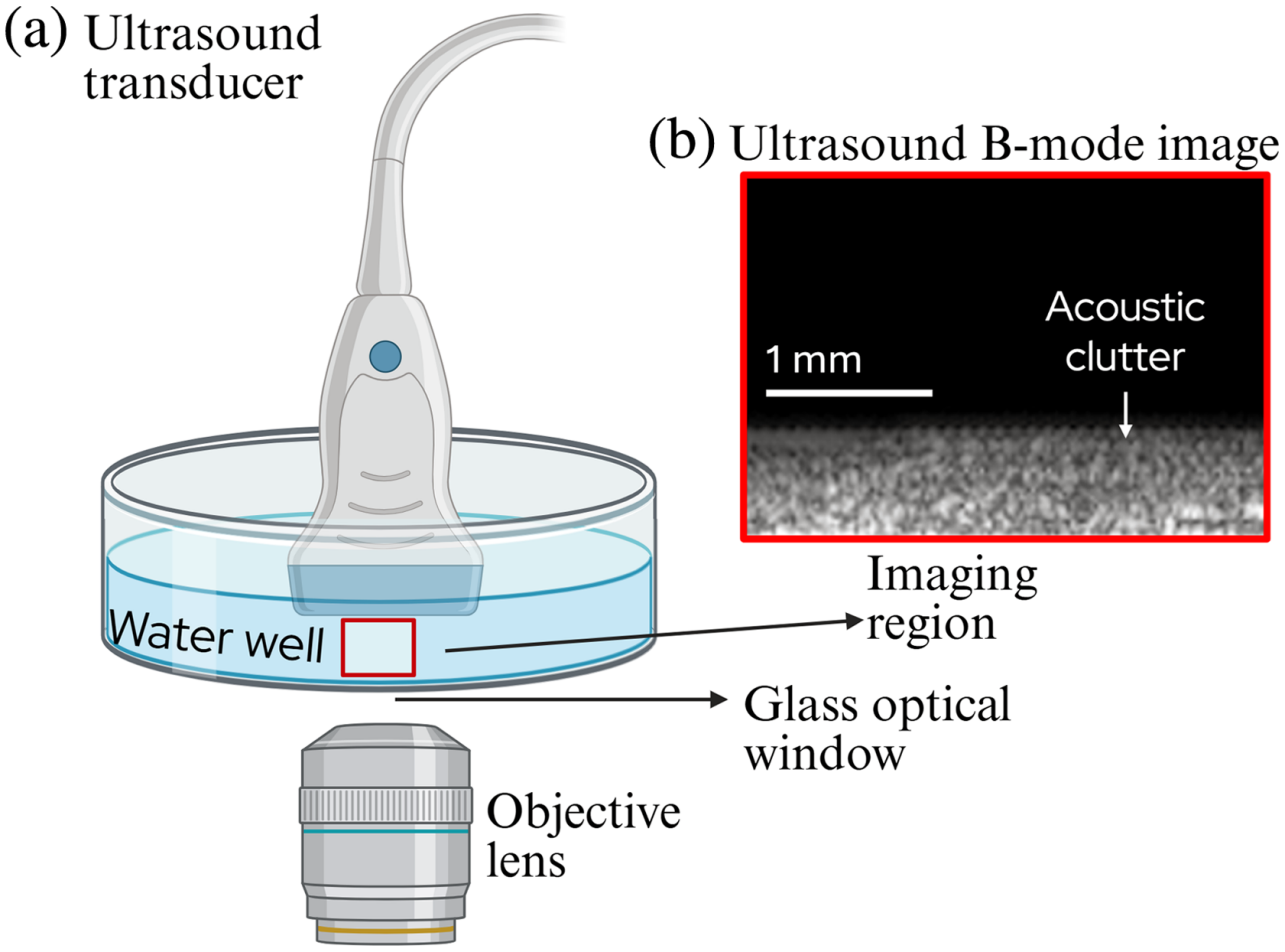
(a) Diagram of the multimodal US and MPM scope setup, with an ultrasound transducer from above and objective lens from beneath. (b) Zoomed in B-mode image of just above the glass optical window depicting a signal that could be detected from the red region in panel (a) from the L22-14 transducer, transmit F/6, receive F/4, 25 V excitation, and a glass optical window. Signal is detected in the anechoic water region right above the glass window. Partially created in BioRender.^11^

In the development of this direct correlated MPM/US scope, previous studies found that the quality of the US images is compromised by the presence of spurious echoes caused by the highly reflective glass optical window used for MPM microscopy in the multimodal scope.^4^ These spurious echoes are commonly referred to as acoustic clutter. This clutter is present in the imaging well no matter what tissue sample was placed in the well and even when there was no tissue sample (only water). To help understand this issue, Fig. 1(a) shows a diagram of the MPM-US multimodal scope. The tissue sample is placed in an imaging well with a glass bottom acting as an optical window, though the tissue sample is not depicted in Fig. 1. Figure 1(b) shows an example brightness mode (B-mode) acquired only with water (anechoic) between the transducer and the optical window. Yet, spurious echoes (clutter) can be clearly detected in the region before the glass window. One source of these unwanted echoes results from a ringing effect caused by the interaction of the ultrasound pulse on the optical window, which acts like a drum. This ringing generates acoustic energy outside of the main axis of propagation (known as off-axis) of the ultrasound pulse. This off-axis intensity, referred to as off-axis clutter, contributes to the visible “haze” shown in Fig. 1(b) and can significantly degrade the quality of the ultrasound images of tissue samples.

To reduce clutter by the glass optical window, Pinkert et al.^4^ compared different ultrasound beamforming approaches by reducing the size of the aperture for both the transmit and receive ultrasound beams, i.e., the numbers of active ultrasound transducer elements during transmission and reception. Beamforming refers to the process of ultrasound beam focusing by customizing the timing and amplitude of the excitation of the elements of the ultrasound transducer, as well as the technique for summation of the detected echo signals. The authors found that a combination of different transmission and reception focal configurations (f/#=6 for transmission and f/#=9 for reception, where f/# is the ratio of the focal distance and the aperture) decreased clutter at the expense of lower signal-to-noise ratio.^4^

In this work, we present a comprehensive solution to mitigate acoustic clutter in the multiscale MPM/US scope that combines alternative optical window materials with advanced beamforming techniques, both without compromising the optical image quality. To this end, first, we investigate a collection of low-acoustic reflectivity materials commonly used in optics research to be used as optical windows. Then, we investigate the use of advanced beamforming based on synthetic focusing to reduce the incident acoustic power on the optical window. The compromise on optical image quality is evaluated by quantifying the MPM component’s point spread function (PSF) when the alternative optical windows are used. Based on our interest in developing ultrasound techniques sensitive to collagen fiber morphology,^9^^,^^12^^–^^14^ we compare the effect of different window materials on the automatic quantification of morphological metrics of collagen fiber alignment and density evaluated in second harmonic generation (SHG, a MPM imaging mode well suited for imaging collagen) images of rat tendon. Our main finding is a definite solution to the detrimental effect of acoustic clutter by identifying a combination of a low-reflectivity window material with a reception beamforming f/# of f/2 that reduces the acoustic clutter in brightness-mode (B-mode) images by a least 2 dB without introducing errors larger than 5% in automated SHG-based collagen fiber alignment estimation. This will pave the way for future studies investigating the correlation of collagen fiber morphology with various ultrasound-based contrast modes.

## 2 Methods

### 2.1 Selection of Alternative Optical Windows

A set of inclusion criteria was defined to select alternatives to the glass optical window:

1. The material should be a polymer/plastic used in the manufacturing of optics parts or used in optics research.
  a. This ensures that the material is optically clear. This also ensures that the material possesses a lower acoustic impedance relative to glass because polymers/plastics have a lower sound speed and density compared with glass.
2. The polymer/plastic must be manufactured as a thin film (120 to 220  μm) and be available to purchase.
  a. Most high-numerical aperture (NA) microscopy objective lenses used in imaging such as MPM are designed to have 170-μm of glass in between the lens and the tissue sample. To achieve the best optical quality, the optical window should be 120 to 220  μm thick. Furthermore, commercial availability ensures accessibility.
3. The film must be optically favorable for MPM.
  a. This is a general qualitative assessment of optical clarity. A material that can be used well in optics lenses may not work well optically as a thin film. For example, polystyrene thin films are not clear, though polystyrene passes the first two criteria.

RP Photonics, an online encyclopedia for optics, was searched to identify manufacturers of optically compatible polymers and plastics.^15^ The websites of these suppliers were reviewed to identify materials that satisfied the inclusion criteria. The output of this search is provided in the “Code and Data Availability” section. An additional proprietary polymer material called Ibidi^®^ polymer (Ibidi, Munich, Germany) was also considered based on its widespread use in optical imaging research.

### 2.2 Acoustic Characterization of Polymer Films for Optical Window

A pulse-echo, reference-based immersion technique was used to measure the reflectivity of the plastic films found using the inclusion criteria described above. This method is also used to measure the acoustic attenuation of the films. Materials with higher attenuation would reduce the reflection from the optical window/air interface on the side of the objective lens, further reducing acoustic clutter. In this method, the reflectivity of the material of interest was obtained by comparing it to that of a planar reflector with known reflectivity. This method is described in Sec. S.1 in the Supplementary Material. A broadband 30-MHz single-element immersion transducer (30  MHz−6  dB bandwidth, 0.25″ aperture, 0.75″ focal length, V375-SU, Olympus, Waltham, Massachusetts, United States) was connected to a UT340 pulse-receiver (Utex, Ontario, Canada). The transducer was excited with a 100-V, 2.00-ns width pulse, and the reflected echo was amplified with a 25-dB gain. The reflected radiofrequency (RF) signal was transferred to a WaveSurfer 4024HD oscilloscope (Teledyne LeCroy, Chestnut Ridge, New York, United States) for further analysis. A plexiglass planar reflector (amplitude reflection coefficient $\Gamma_{0}=0.37$) was placed at the focus of the transducer and perpendicular to the ultrasound beam to obtain a reference signal of the ultrasound pulse. Then, the plexiglass reflector was replaced by the thin film, placing the front face of the film (transducer facing side) at the focus. Data were acquired for five independent locations spaced 1 mm apart on the planar reflector and the sample film. For each location on the film or reflector, a sequence of 500 pulse-echo waveforms was recorded.

Four acoustic properties of the samples were estimated from the RF echo signals of the samples and the reflector: the amplitude reflection coefficient $\Gamma_{s}$, the characteristic acoustic impedance $Z_{s}$, and the slope ($\alpha_{s}$) and 30-MHz intercept ($\alpha_{30}$) for the best fit attenuation curve $\alpha (f)=\alpha_{s}(f-30)+\alpha_{30}$. First, power spectra $S_{\mathrm{ff}}(f)$ and $S_{\mathrm{bf}}(f)$ from the echo signals from the front face and back face of the sample, respectively, were obtained by windowing the RF echoes with 0.115-μs rectangular windows and obtaining the squared amplitude of the Fourier transform. The average power spectrum of the planar reflector $\left|\overset{¯}{S_{0}(f)}\right|$ was obtained similarly, and the upper bar indicates the average from five independent measurement locations over the face of the reflector.

The estimator for the magnitude of the sample reflection coefficient is

$$|\Gamma_{s}|=|\Gamma_{0}|\frac{\left|S_{\mathrm{ff}}(f_{c})\right|}{\left|\overset{¯}{S_{0}(f_{c})}\right|},$$ (1)

where $f_{c}=30\;\mathrm{MHz}$, the center frequency of the transducer. The acoustic impedance was then estimated from the amplitude reflection coefficient

$$Z_{s}=Z_{w}\frac{1+|\Gamma_{s}|}{1-|\Gamma_{s}|},$$ (2)

where $Z_{w}$ is the characteristic impedance of water. Lastly, the frequency-dependent attenuation function was estimated by fitting the attenuation versus frequency data to a line. The linear model for attenuation was $\alpha (f)=\alpha_{s}\times (f-30)+\alpha_{30}$, where α and $\alpha_{30}$ are in units of $\mathrm{dB}\;{\mathrm{cm}}^{-1}$ and $\alpha_{s}$ in units of $\mathrm{dB}\;{\mathrm{cm}}^{-1}\;{\mathrm{MHz}}^{-1}$. $\alpha_{30}$ is the attenuation at the center frequency of 30 MHz. The slope $\alpha_{s}$ and constant $\alpha_{30}$ were estimated from a linear least-squares fit of the following data:

$$(f,8.686\times \ln(\frac{|S_{\mathrm{bf}}(f){|}^{2}}{|S_{\mathrm{ff}}(f){|}^{2}}\times \frac{1}{(1-\Gamma_{s}^{2}{)}^{2}})\times \frac{1}{-4d}),$$ (3)

where the frequency f is restricted to the −3-dB bandwidth of the reflector power spectrum (19 to 34 MHz), and d is the thickness of the sample in centimeters. If the sample has a different reflection coefficient on the front face versus the back face, then the slope $\alpha_{s}$ and constant $\alpha_{30}$ were estimated from a linear least squares fit of the following data:

$$(f,8.686\times \ln(\frac{|S_{\mathrm{bf}}(f){|}^{2}}{|S_{\mathrm{ff}}(f){|}^{2}}\times \frac{\Gamma_{\mathrm{ff}}^{2}}{(1-\Gamma_{\mathrm{ff}}^{2}{)}^{2}\Gamma_{\mathrm{bf}}^{2}})\times \frac{1}{-4d}).$$ (4)

At each of the five lateral locations, a single estimate of the four properties was obtained using the average spectrum from the 500 waveforms run through the estimators Eqs. (1)–(4). The mean and standard error were then calculated from the five lateral locations. Section S.1 in the Supplementary Material presents the derivations of Eqs. (1)–(4).

### 2.3 Characterization of Acoustic Clutter Under Different Beamforming Strategies

**Fig. 2:**
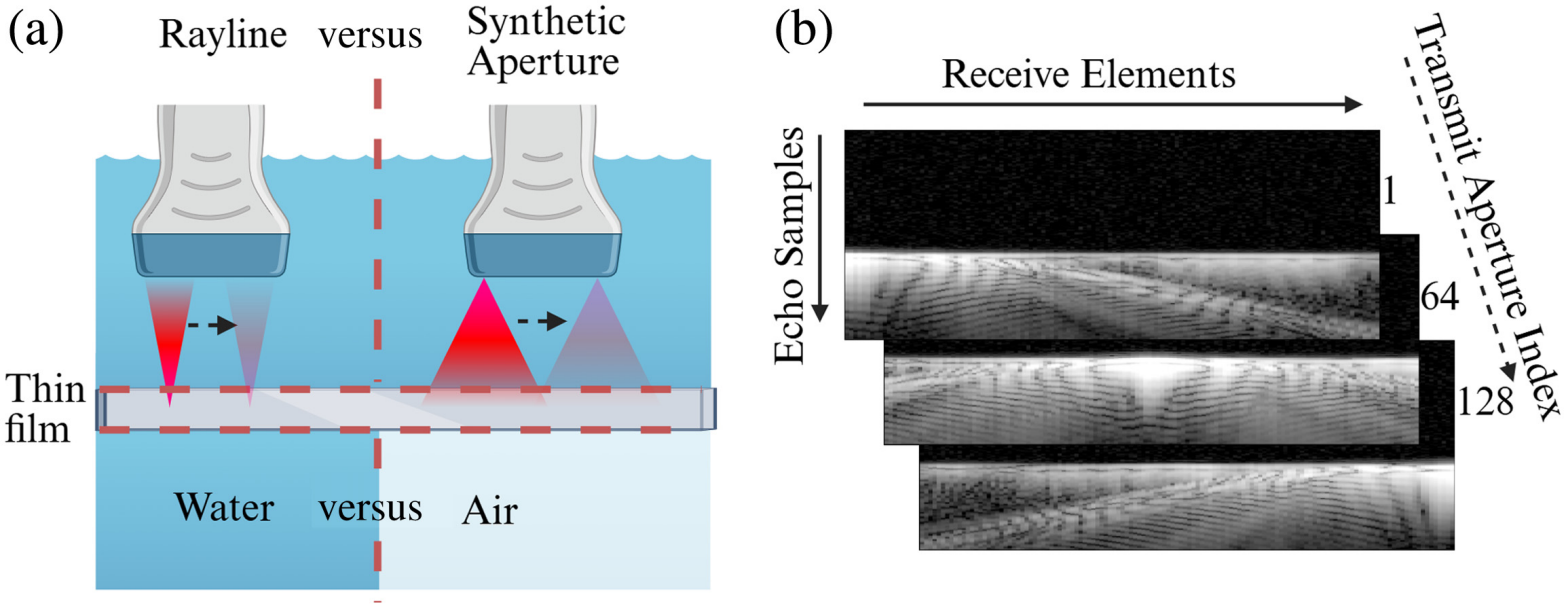
(a) Diagram illustrating the various beamforming and underside coupling conditions for the acoustic clutter experiments. The black arrow indicates the direction in which the acoustic beam is scanned across the sample. (b) Echo amplitude samples are stored in a 3D FRM, where the first dimension represents the transmit aperture index, and the other two dimensions represent the receive element and echo samples. The example FRM frames shown are from a glass/dry/rayline scan. Created in BioRender.^18^

Acoustic clutter caused by the ringing of the optical window was measured for different optical window materials, different transmit beamforming strategies, and different media (air or water) placed on the face of the optical window furthest from the transducer, referred here to as the back face. These variables are expected to affect the ringing effect as follows:

1. Optical window material: The acoustic radiation force of the ultrasound pulse interacting with the front face of the optical window is hypothesized to be a source of the ringing. The use of optical windows with lower acoustic reflection coefficients would decrease the acoustic radiation force incident on the front face (transducer side) by allowing a larger portion of the acoustic energy to transmit through the front face. The ultrasound pulse transmitted will be attenuated by the optical window material before interacting with the back face of the window, where acoustic radiation force on the back face of the window is also hypothesized to be a source of the ringing. Thus, a low reflection coefficient and a high attenuation coefficient are expected to reduce the ringing of the optical window.
2. Beamforming techniques: Beamforming techniques that result in lower acoustic intensity incident on the window would reduce acoustic radiation force on the optical window, thus reducing the ringing effect. Two beamforming techniques were compared: rayline scan acquisition (with physical transmission focus) and a synthetic aperture acquisition (with synthetic transmission focus). A rayline scan, the default beamformer on the MPM/US scope, uses delayed excitations applied to a subset of the transducer’s piezoelectric elements to create a physically focused ultrasound pulse that will interact with the optical window.^7^ A synthetic aperture sequence pulses a single element at a time,^16^ resulting in weaker, unfocused ultrasound pulses interacting with the optical window. The received RF echo signals for all single-element excitations are then processed to reconstruct a synthetic rayline scan.^17^ The weaker, unfocused beams from the synthetic aperture scan are expected to cause less ringing, which is why this beamformer is investigated. See Fig. 2 for a diagram demonstrating these two ultrasound transmission strategies.
3. Medium placed underneath the optical window: As mentioned, the acoustic radiation force from the reflection off the back face of the optical window may also cause the ringing. Under default imaging conditions, air is the medium underneath the imaging well. The optical window/air interface has a large reflection coefficient (close to 100%), so placing water underneath the well will drop the back face reflection coefficient to the optical window/water reflection coefficient. These two conditions (water- or air-underneath) are referred to as “wet” or “dry,” respectively. Diagrams in Fig. 2 with labels “water” and “air” illustrate the “wet” and “dry” conditions, respectively.

To collect data for every imaging combination of optical window/transmit beamformer/back face coupling (wet versus dry), the glass bottom of Cellvis D60-30-1.5N dishes used as optical windows was replaced with the different polymer films. The polymethyl pentene film had a matte-finish side that was placed on the ultrasound side (see Sec. S.2 in the Supplementary Material). The wells with the new windows were placed on the motion stage of the MPM/US scope and filled with water. The ultrasound transducer (L22-14v, Verasonics, Kirkland, Washington, United States) was aligned with the aperture perpendicular to and 8 mm away from the optical window. The time-gain control was set to 150 (ranges from 0 to 1023) for all depths, which will be referred to as the “low-gain” setting, to prevent the high amplitude reflected echoes from saturating the detector.

**Fig. 3:**
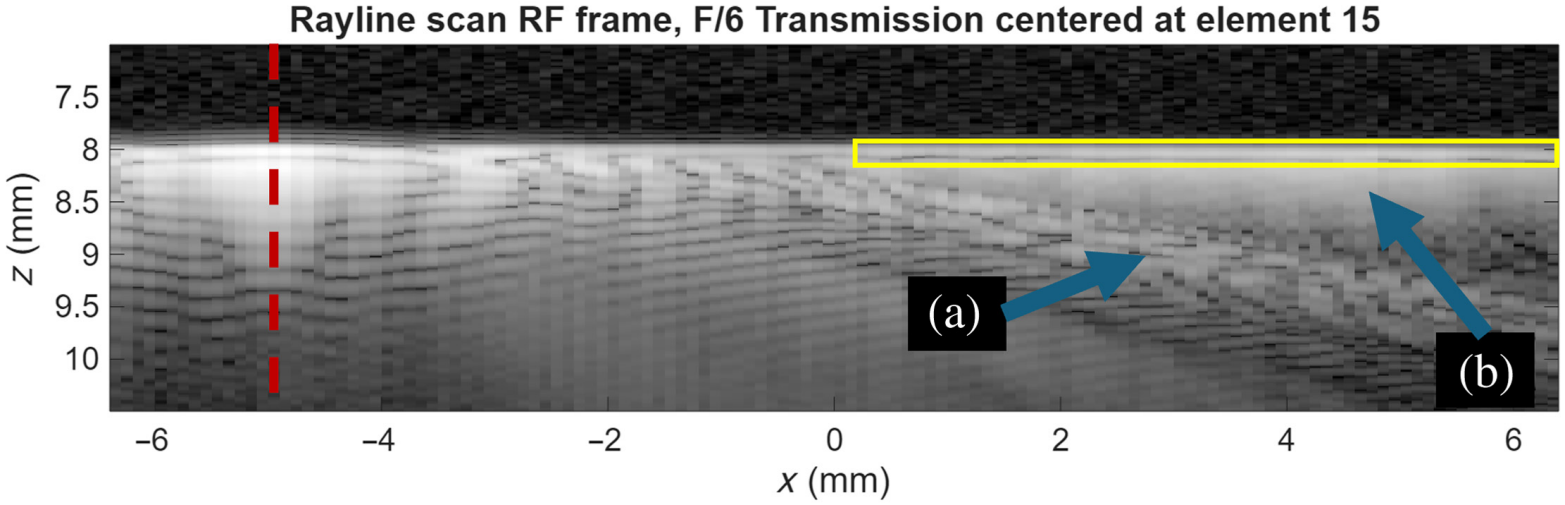
RF frame from a rayline scan aperture with transmission aperture index 15, with the red dashed line representing the rayline axis and the yellow box representing an example ROI to analyze intensity from optical window ringing. (a) Expected reflection from the incident ultrasound pulse. (b) Unexpected off-axis intensity due to the optical window ringing.

For the wet coupling conditions, a small layer of water was placed underneath the imaging well to provide acoustic coupling on the back face of the optical window. For each of the wet and dry conditions, two imaging sequences were collected: first, a rayline scan with a f/6 transmission (13 active elements, focused at 8 mm), and second, a synthetic aperture sequence with single-element transmission. The data from each of the transmissions are processed differently, and these will be described below:

1. For the rayline scan, each of the 128 f/6 transmissions is indexed such that the transmission index corresponds to the center element of the 13-element active aperture. For example, rayline transmission #10 corresponds to the f/6 transmission using elements [4, 5, 6, 7, 8, 9, 10, 11, 12, 13, 14, 15, and 16] because element 10 is the center element of the active aperture. Apertures at the elements near the edge of the transducer needed to be shortened, so rayline transmission #3 consists of only elements [1, 2, 3, 4, 5, 6, 7, 8, and 9]. For each transmission event, all 128 elements sample the backscattered echo amplitude at a sampling rate of 62.5 MHz for 28.656  μs, resulting in 1792 samples. All the data from the rayline scan are packaged into a 128×128×1792 3D matrix in MATLAB, called the focused response matrix (FRM), where the first dimension corresponds to the f/6 transmit aperture index; the second dimension corresponds to the single element recording the echoes, and the third dimension holds the time-series samples of the backscattered ultrasound echoes. Lastly, a Hilbert transform is performed on the time-series samples to recover the analytic signal. The time-series sample is also often represented as spatial samples using the transformation $d=\frac{t\times c}{2}$, where c is the speed of sound of water (1480  m/s). For a given f/6 transmission event i, the 2D slices along the first dimension [FRM(i,:,:)] shown the response of the 128 elements when transmission aperture i is used. We call this slice an RF frame, resulting in 128 RF frames stored in the FRM. Three RF frames are shown in Fig. 2(b), and the organization of the FRM is diagrammed. In addition, the RF frame from transmission index 15 is shown in Fig. 3.
2. For the synthetic aperture data, a similar 128×128×1792 3D matrix is constructed. As only a single element is pulsed at a time, the first dimension of the matrix holds the transmitting element number, the second dimension holds the receiving element number, and the third dimension holds the sampled backscattered ultrasound echoes. A Hilbert transform is again applied to the time series samples to recover the analytic signal. This matrix is called the inter-element-response matrix (IRM) because this matrix holds the response from every pair of transmit/receive elements. Similar to the procedure outlined by Holmes et al.,^17^ slices along the first dimension of the IRM are delayed and summed to transform the IRM to a synthetically constructed FRM, which provides the reconstructed response to all 128 f/6 transmissions. This synthetic aperture FRM is organized the same as the rayline FRM. Section S.3 in the Supplementary Material demonstrates equivalency of the physical and synthetic reconstructions.

Acoustic clutter was quantified at two levels in the ultrasound reconstruction process: pre-receive beamforming (where clutter in the RF frames is measured) and post-receive beamforming (where clutter in the B-mode images is measured). For the pre-receive beamforming clutter measurements, analysis is performed on the RF frames found in the rayline FRM and synthetic aperture FRM. For each optical window/transmit beamformer/back face coupling combination, a region of interest (ROI) was defined for each RF frame. The ROI was placed at the depth of the optical window and far enough away from the center of the transmission axis used for the RF frame that the expected wavefront was not included in the ROI, which was found to be >5  mm away on the x-axis (see Fig. 3). To account for the variations in signal values due to differences in mathematical reconstructions between rayline and synthetic aperture, the RF frames were scaled to account for the different noise levels of the two beamforming strategies. The noise level for the SA transmit reconstruction was measured by averaging 10 times the log compressed squared envelope of the FRM matrix where the transmission index matches the receive index and in a region from 1 to 6 mm deep (anechoic water). This was done for the middle 81 RF frames. In mathematical notation, the estimated noise floor can be expressed as $\frac{1}{81}\overset{i=104}{\underset{i=24}{\sum }}\text{mean}(20\times \log\;10(|\mathrm{FRM}(i,i,1\;\mathrm{mm}:6\;\mathrm{mm})|+1))$. The measurements of noise were repeated for the rayline scan RF frames.

After scaling all the FRMs, the maximum value of the 20 × log 10 compressed envelope of the RF frames was measured in each frame’s ROI. Only the RF frames generated with the central 81 transmission events were considered in the estimation of the mean and standard deviation so that the partial-aperture effects when transmitting close to the edge of the transducer were avoided. The average values of the maximum squared envelope signal were normalized to the average off-axis squared envelope signal from a rayline scan of a glass optical window. As the squared envelope was normalized, it can be considered a comparison of acoustic intensity relative to the glass-rayline off-axis intensity. A one-sided tail t-test of independence was performed to determine whether the off-axis clutter mean intensities were significantly different from the glass/rayline scan/dry condition (with p≤0.05). Another one-sided tail t-test of independence was performed on each material to determine the statistical significance of differences between rayline scanning and synthetic aperture (with p≤0.05). Both tests used the function “ttest2” in MATLAB (R2020b, MathWorks, Natick, Massachusetts, United States).

To quantify the final effect of clutter on the post-receive-beamformed data, dynamic receive focusing with delay-and-sum reconstruction was implemented on the RF echo signals acquired from all combinations of optical window/transmit beamforming/back-face coupling, as described by Wagner et al.^7^ Sec. II. Similar to the pre-receive beamforming analysis above, the noise levels differ between synthetic aperture B-mode images and the rayline B-mode images, as well as for different receive beamforming f/#. Thus, for each f/#, and for the SA and rayline beamformed B-modes, the noise levels were measured by averaging the B-mode values in the region 1 to 6 mm deep, averaging across the middle 81 A-lines, and averaging across the different material B-modes. For each receive f/#, the synthetic aperture beamformed data and the rayline beamformed data were scaled to match the respective noise floors at 0 dB. To quantify the effect of off-axis clutter on the post-receive-beamformed image, the average dB value of the B-mode images was measured within a 0.45 mm (axial) × 4 mm (lateral) ROI with the lower border 0.15 mm above the front face of the optical window. This region was chosen because the images do not show any clutter beyond 0.6 mm above the optical window, whereas locations closer than 0.15 mm are affected by the main echo of the optical window. Following the study by Pinkert et al.,^4^ the effect of off-axis clutter was investigated for different receive f/# values. For the f/# that shows the greatest decrease in acoustic clutter, one-sided tail Student t-tests were performed using MATLAB function ttest2 to determine whether the levels of acoustic clutter were significantly lower than those obtained with the state of the art (glass-rayline-dry acquisition) with the same receive f/#. Statistical significance using MATLAB’s ttest2 function was also investigated, comparing the different acquisition conditions for each material.

### 2.4 Optical Point Spread Function for Different Optical Windows

The MPM scope is based on an SHG configuration and consists of a laser light source given by a 3.5-W femtosecond titanium:sapphire laser (Chameleon Ultra II, Coherent, Santa Clara, California, United States). The beam-path includes x–y galvanometer mirrors (62xxH series, Cambridge Technology, Bedform, Massachusetts, United States) allowing for fast laser-scanning imaging. The objective lens used is a 20× magnification 0.75 NA air objective (Plan Apo VC, Nikon, Tokyo, Japan). A 3D motorized stage holds the sample slide or imaging well. A detailed description of the scope can be found in the original publication by Pinkert et al.^2^

The optical PSF follows the procedure by Cole et al.,^19^ which involves imaging sub-resolution fluorescent beads and estimating the full-width at half-maximum (FWHM) of the resulting peak SHG signals of each bead. Fluoresbrite^®^ fluorescent yellow-green, 0.2-μm glass beads (Polysciences, Warrington, Pennsylvania, United States) were mounted with Cyctoseal-60 mounting medium (Thermo Fisher Scientific, Waltham, Massachusetts, United States) onto the various optical windows being investigated. The fluorescent bead solution was highly diluted to create a sparse distribution of beads, making it easier to locate a single bead instead of a cluster of beads. At least three isolated beads were found for each optical window. Using a depth spacing of 0.2  μm, a z-stack of SHG scans was taken that covered all depths where the bead signal was present. The z-stacks were opened in FIJI^20^ (a distribution of ImageJ), and all the z-planes were summed. Bead profiles in the x and y directions were extracted as comma-separated-value (csv) files and loaded into MATLAB (R2020b, MathWorks, Natick, Massachusetts, United States), where the FWHM was estimated by finding the difference between the first and last locations where the signal was greater than half of the maximum value of the bead signal. The median was calculated in MATLAB using the built-in “median” function, and the range was calculated by taking the difference between the maximum and minimum using the “max” and “min” MATLAB built-in functions. The MATLAB scripts for estimating the PSF are available, as described in the “Code and Data Availability” section.

When following the PSF protocol for the thin film made from polymethyl pentene, which has a matte finish on one of the sides, the beads and mounting media were placed on the matte-finish side. As demonstrated in Sec. S.1 in the Supplementary Material, placing the matte finish in contact with the mounting medium significantly improves optical image quality.

### 2.5 Effects of Optical Window Material on SHG Images of Collagen Fibers

Collagen, the most abundant protein in the human body, plays a crucial role in the structural integrity and function of various tissues. Its hierarchical organization, from fibrils to fibers, imparts specific mechanical properties that are vital for tissue function and health.^21^ Collagen fibers exhibit autofluorescence which can be imaged label-free with SHG microscopy. The spatial resolution of SHG microscopy can be less than 1  μm, which is ideal for imaging collagen fibers that range in diameter from 1 to 40  μm. These collagen fibers are intertwined in complex collagen networks whose morphological features (fiber density, alignment, and cross-linking) can vary during physiological or pathophysiological processes and can be quantified from SHG images through automated processing algorithms.^22^^,^^23^ Moreover, collagen fibers are shown to have a large acoustic-scattering cross-section and have been proposed as a major source of echo signals in soft tissue.^24^ Thus, the proposed multiscale scope is well-suited to study the association between collagen fiber characteristics and their effect on ultrasound signals. Key to this task is the ability to accurately describe the collagen fiber network morphology when imaged through the alternative optical windows required to obtain high-quality ultrasound data.

Motivated by this use case, we also investigated that the use of alternative optical window materials did not significantly compromise the morphological description of collagen fiber on SHG images quantified through CurveAlign.^22^^,^^23^ CurveAlign quantifies the angle, alignment, and density of collagen fibers through the use of a curvelet transform. The accuracy of CurveAlign’s quantitative estimates of collagen fiber features is crucial to the eventual development of ultrasound-based techniques sensitive to collagen fiber features. The following test was conducted to determine the relative error in CurveAlign quantitative alignment estimates of a rat tail tendon when a coverslip material other than glass was used.

**Fig. 4:**
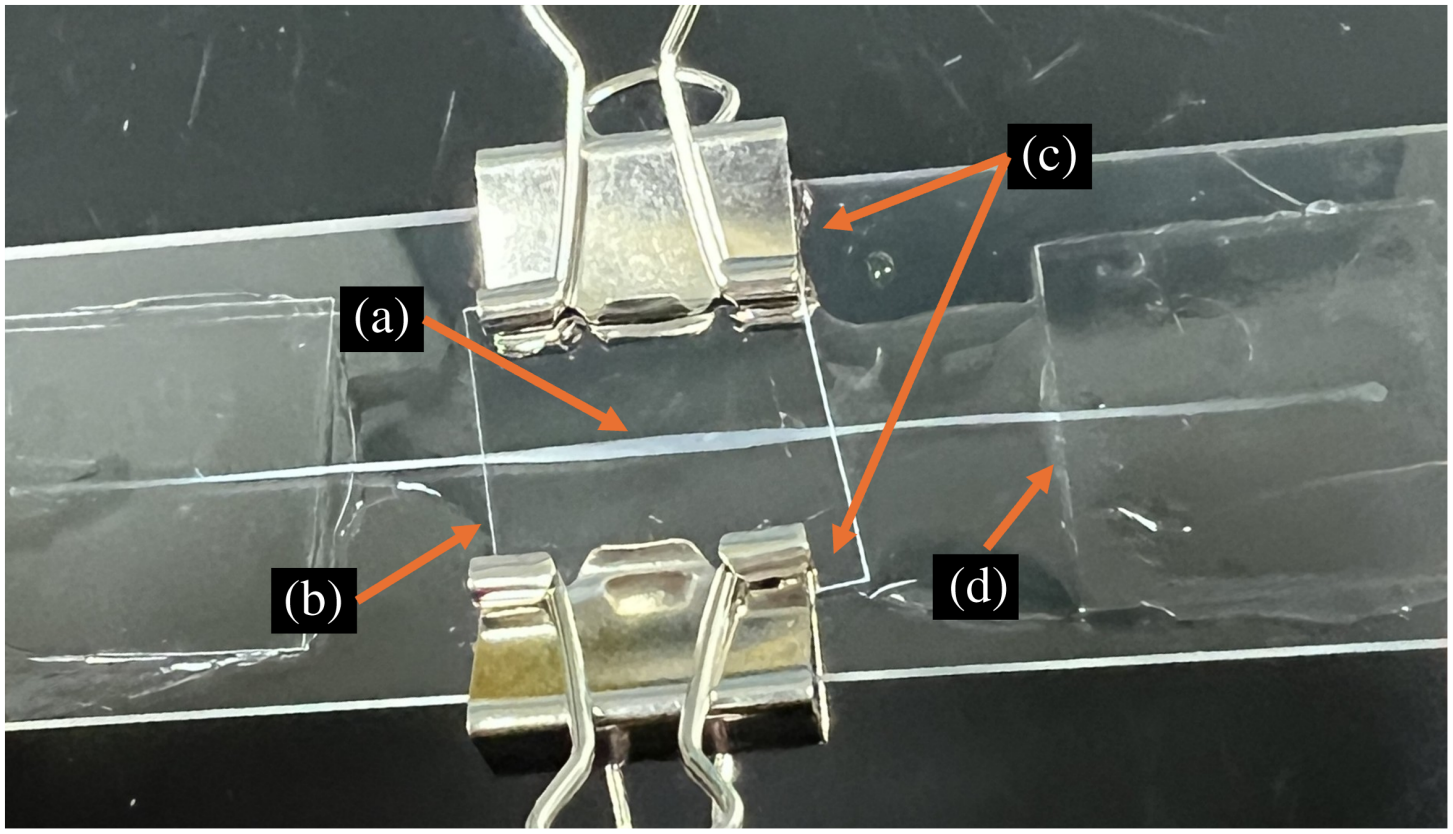
Example rat tail tendon slide with removable coverslips held by binder clips. (a) Rat tail tendon, at the center point where the objective lens would image. (b) Removable coverslip, glass coverslip for this image. (c) Small binder clips. (d) Glued glass coverslips.

The rat tail tendon was extracted following the procedure outlined by Bruneau et al.^25^ The tendon was trimmed to be 6.5 cm, just shorter than the length of the glass slide (Fisherbrand Microscope Slides, Fisher Scientific, Pittsburgh, Pennsylvania, United States). Glass coverslips (Fisherbrand Microscope Cover Glass #1.5, 22  mm×22  mm, Fisher Scientific) and Cytoseal-60 were used to affix the ends of the tendon, applying slight tension to ensure that the tendon’s fibers were highly aligned. A small coverslip made with the different optical window materials was held to the center portion of the tendon using generic small binder clips, with deionized water placed in between the glass slide and coverslip to ensure good contact. Figure 4 shows an example of this slide. The middle coverslip can be removed and replaced by a coverslip of another material, allowing for the same rat tail tendon segment to be imaged under the different optical window materials (Fig. 4). For the material polymethyl pentene, which had a matte finish on one of the sides, the matte finish was in contact with the rat tendon and the deionized water to reduce optical scattering from the matte finish, as described in Sec. S.1 in the Supplementary Material.

To image the slide, four 128  μm×128  μm SHG images were taken in a 4×1 grid. Each image was the average of three images (1024×1024 resolution and zoom 2). The four images were processed by CurveAlign, using 3% of curvelet transform coefficients. The median and range were calculated from these four CurveAlign estimates, again using the MATLAB built-in functions “median,” “max,” and “min.”

### 2.6 Comparison of Ultrasound Image Quality of Fabricated Collagen Hydrogels

It is important to be able to detect low-echogenic scattering sources in the presence of electronic noise and clutter sources. Two 2-mg/mL collagen hydrogels were fabricated using the instructions found in application note 26 by Ibidi^®^.^26^ Each gel was 15 mL in volume, which resulted in a gel a little thicker than 1.5 mm when poured into the Cellvis D60-30-1.5N wells. One well was unaltered (glass optical window), and one well had a cyclin olefin copolymer (COC) optical window. For each collagen gel, a synthetic aperture acquisition and a rayline acquisition were performed and B-mode images reconstructed, following the same data acquisition steps found in Sec. 2.3. The two differences in the acquisition here are that no wet conditions were investigated, and the gain in the time-gain control on Verasonics was maximized for all depths, which will be referred to as the “high-gain” settings. This allowed visualization of the speckle pattern generated from scattering within the collagen gels. In addition, a 3D US dataset of each collagen gel was collected following the procedure outlined in Ref. 3, but with receive f/#s of 1 and 2.

The respective noise floors for the synthetic aperture and rayline acquisitions were measured by averaging the log compressed squared envelope for the middle 41 A-lines in a water-only region. The dB scales in the B-mode images were then scaled to have the noise floor be 0 dB across reconstruction methods. 3D SHG data were acquired for each gel. The data took an x–y SHG acquisition that was 3  mm×0.256  mm in size at 51 z-depths that were spaced 10  μm apart. The US and SHG data were registered following the protocol outlined in Ref. 3. Both x–y and x–z plane slices of the 3D registered SHG and US data were taken and overlaid to show the effect and extend of the clutter above the optical window. The x–y slice was extracted at z=0.4  mm above the optical window. The x–z slice corresponded to the middle slice in the y-dimension of the 3D SHG data.

## 3 Results

### 3.1 Selection of Alternative Optical Windows

Table 1 lists all the materials that were identified through the RP photonics search and the inclusion criteria they fulfill. The four criteria were met by polycarbonate (PC, 8010, Lexan, Miami, Florida, United States), polymethylmethacrylate (PMMA, No. 9011-14-7, Goodfellow, Pennsylvania, United States), cyclic olefin copolymer (COC, 240  μm thickness, Topas grade 8007, Polyplastics USA, Inc., Farmington Hills, Michigan, United States; COC, 175 um thickness, no. 10001999, Microfluidic Chipshop, Jena, Germany), polymethyl pentene (PMP, TPX-DX-845, Goodfellow, Pennsylvania, United States), and polyethylene terephthalate (PET, No. 462, Melinex, Chester, Virginia, United States). This list was complemented with Ibidi^®^ (μ-Dish 35 mm, high, Ibitreat, Ibidi, Gräfelfing, Germany), which is commonly used as an optical window in cell imaging. All materials found were within 175±10  μm, except for PMP which was 125  μm and too thin for acoustic characterization. For this reason, we used a thicker 250-μm PMP sample.

**Table 1:** Results of the inclusion/exclusion criteria for determining potential optical window materials.

	Used in optics for lenses, found on websites	120 to 220 μm thick	Optically clear thin film
Glass	☑	☑	☑
Polycarbonate	☑	☑	☑
Polymethyl methacrylate	☑	☑	☑
Cyclin olefin copolymer	☑	☑	☑
Polyethylene terephthalate	☑	☑	☑
Polymethyl pentene	☑	☑	☑
Polyether sulphone	☑	☑	☒ (low transmittance for 445-nm light)^27^
Polyetherimide	☑	☑	☒ (amber colored)
Polystyrene	☑	☑	☒ (not transparent)
Polyurethane	☑	☒	☒
Acrylonitrile butadiene styrene acrylon	☑	☒	☒
Styrene-acrylonitrile copolymer	☑	☒	☒
Styrene methyl methacrylate	☑	☒	☒
Cyclic olefin polymer	☑	☒	☒

PMP thin films could only be acquired from manufacturers with a matte finish on one side of the film. A preliminary evaluation of SHG imaging of fluorescent glass beads (Sec. S.1 in the Supplementary Material) indicated that acceptable image quality could be achieved by placing the matte finish in contact with the sample/water. This configuration was used in the imaging tests.

### 3.2 Acoustic Characterization of Polymer Films for Optical Window

**Fig. 5:**
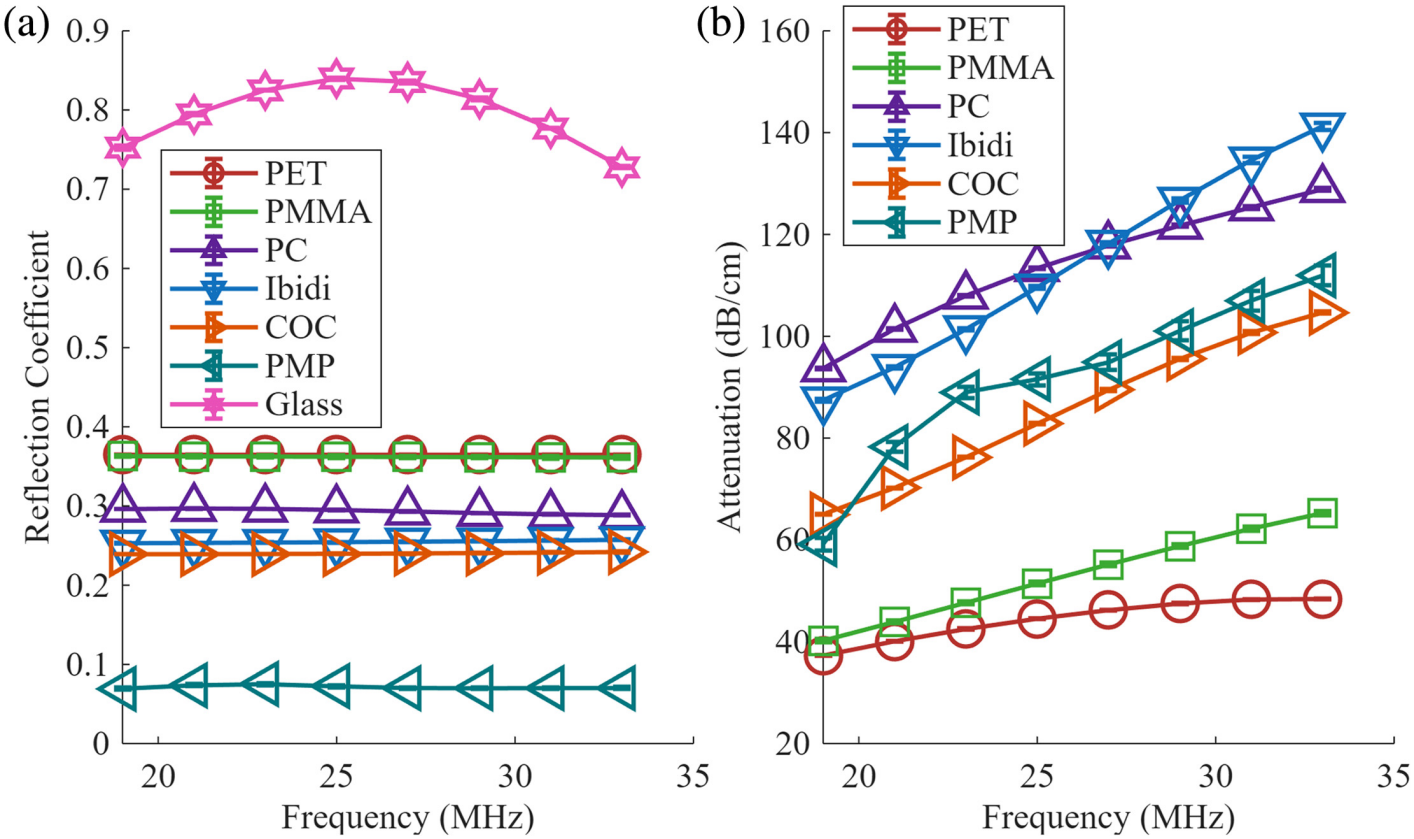
Reflection coefficient (a) and attenuation (b) as a function of frequency of investigated polymers used as potential optical windows. Error bars represent the standard error of the measurements from five independent locations on the material. Error bars may be hard to see because some estimates have very small errors.

All optical window materials showed reflection coefficients that were less than 50% that of glass. Reflection coefficients and attenuation for different optical materials are plotted in Fig. 5.

Table 2 summarizes the acoustic properties of the materials used as optical windows. PMP is listed twice, due to observed differences in reflectivity between the matte-finish side and the gloss-finish side of the film. Of the polymers investigated, PMP has the lowest reflection coefficient, and PET has the highest reflection coefficient. Ibidi^®^ and COC had the next lowest reflection coefficients, of 0.26 and 0.24, respectively. Ibidi^®^ had the highest attenuation at 30 MHz ($\alpha_{30}$), with PET having the lowest attenuation.

**Table 2:** Acoustic properties of materials used as optical windows with ± standard errors. Standard error is from the five independent locations measured on the sample face.

	$\left|\Gamma_{s}\right|$ @ 30 MHz	$Z_{s}$ (MRayl)	$\alpha_{s}$ ($\mathrm{dB}\;{\mathrm{cm}}^{-1}\;{\mathrm{MHz}}^{-1}$)	$\alpha_{30}$ ($\mathrm{dB}\;{\mathrm{cm}}^{-1}$)
Glass	0.797 ± 0.001	13.09 ± 0.08	NA	NA
COC (240 μm)	0.2408 ± 0.0004	2.417 ± 0.002	2.92 ± 0.02	98.3 ± 0.3
Ibidi^®^	0.2560 ± 0.0005	2.498 ± 0.003	3.97 ± 0.06	130.8 ± 0.5
PC	0.2902 ± 0.0002	2.688 ± 0.001	2.36 ± 0.02	123.6 ± 0.2
PET	0.3647 ± 0.0002	3.177 ±0.001	0.751 ± 0.009	48.0 ± 0.1
PMMA	0.362 ± 0.002	3.16± 0.02	1.80 ± 0.04	60.5 ± 0.4
PMP 0.25 mm gloss^a^	0.0822 ± 0.0005	1.744 ± 0.002	3.6 ± 0.2	84 ± 4
PMP 0.25 mm matte^a^	0.0664 ± 0.0009	1.689 ± 0.003	2.8 ± 0.2	123 ± 2

a PMP had different measured reflection coefficients depending on the thickness and surface finish, so the appropriate attenuation estimator was used (see Sec. 2.2).

### 3.3 Characterization of Acoustic Clutter Under Different Beamforming Strategies

**Fig. 6:**
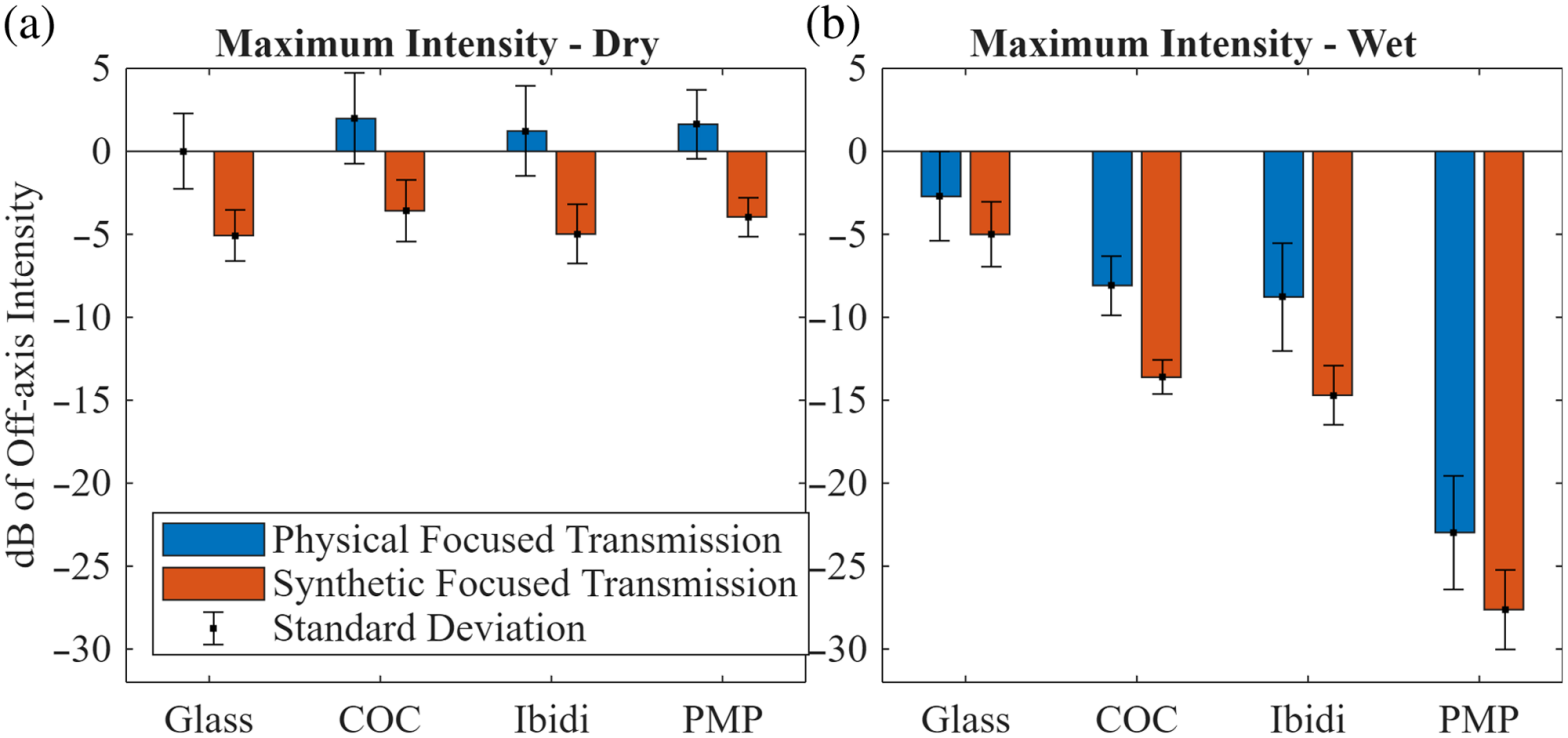
Average intensity from acoustic clutter produced by different material windows and beamforming approaches (physically versus synthetically focused), accounting for the different noise floors and normalized to the glass–rayline off-axis intensity. (a) Dry conditions (SHG-side face of the window on air) and (b) wet conditions (SHG-side face of the window in water). Continuation of this figure found in Fig. S6 in the Supplementary Material.

Different combinations of low-reflectivity optical windows, transmit beamformers, and back-face coupling conditions resulted in different levels of off-axis clutter. Figure 6 shows the maximum off-axis clutter intensity measured in the RF frames for glass and three materials that achieved the greatest pre-receive beamforming clutter reduction under wet conditions. The figure also compares the off-axis intensity between the two beamforming approaches: rayline scanning and synthetic aperture. Not all materials are represented to keep the figures simple yet impactful. The rest of the data for the other investigated materials is found in Fig. S6 in the Supplementary Material. Values are expressed in decibels with respect to the intensity measured with the state-of-the-art configuration: glass optical window, rayline transmit beamforming, and dry back-face coupling. The use of synthetic aperture transmit beamforming (where weak, unfocused beams interact with the optical window) resulted in significant reductions of off-axis clutter intensities for all window materials and under dry and wet back-face coupling conditions with respect to the original glass/rayline beamformer/dry condition (p<0.05), with the exception of glass/synthetic aperture/wet (p=0.17). Figures 6(a) and 6(b) show the clutter intensity obtained under dry and wet conditions, respectively. The wet back-face coupling further reduced the off-axis intensity. The greatest reductions of off-axis clutter intensity were achieved with PMP/synthetic aperture/wet, followed by COC/synthetic aperture/wet and Ibidi^®^/synthetic aperture/wet.

**Fig. 7:**
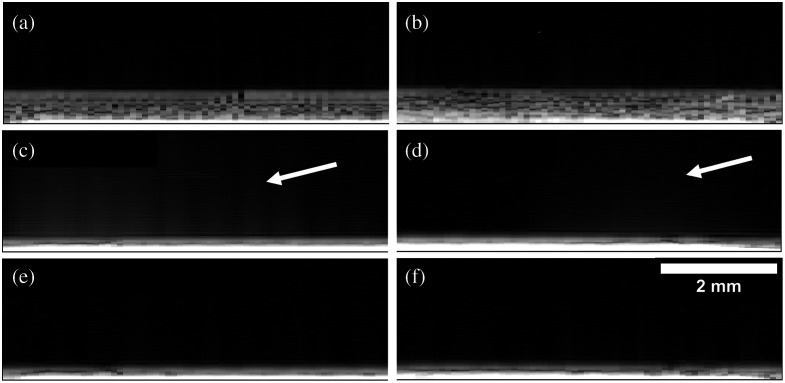
B-mode images with either rayline scan or synthetic aperture scan under dry conditions and different reception beamforming f/#s. Used “high-gain” settings for data collection. Dynamic range of all B-modes is from 0 dB (noise floor) to 60 dB. (a) and (b) Rayline B-mode with f/1 receive. (c) and (d) Synthetic aperture B-mode with f/2 receive. Arrows point to unexpected clutter that does not appear when the low-gain settings are used. (e) and (f) Rayline scan with f/2 receive.

Figure 7 shows example B-mode images of the water/optical window interface for COC and glass and combinations of transmit beamforming approaches (rayline versus synthetic aperture) and receive focusing f/#s. The haze above the windows due to off-axis clutter is clearly visible when a receive f/# of 1 is used [Figs. 7(a) and 7(b)], presumably because a wider aperture detects more of the off-axis intensity. When using synthetic aperture [Figs. 7(c) and 7(d)], there is clutter present that is not found when a lower gain setting is used and is not uniform across the image [white arrows, Figs. 7(c) and 7(d)].

**Fig. 8:**
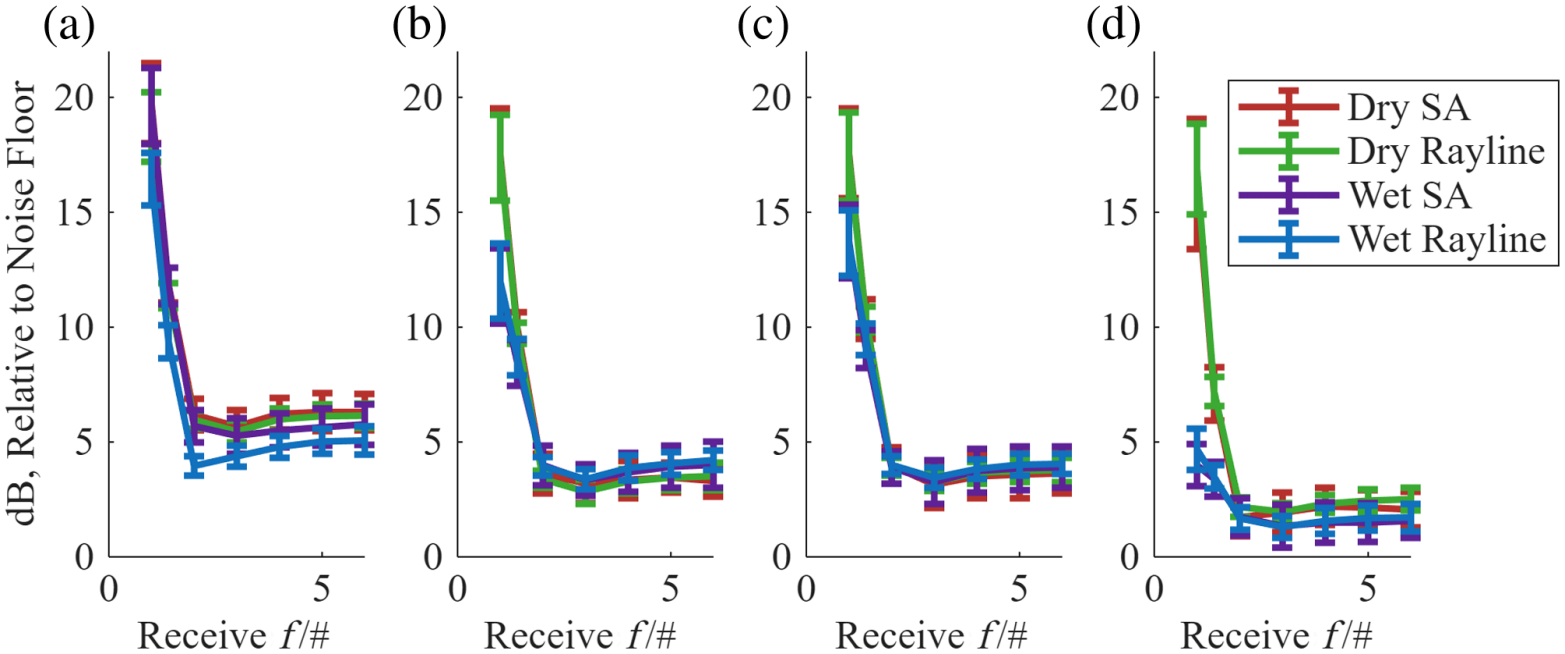
Organized by materials, comparing acquisition conditions. The mean values for the average intensity of signal in the region 0.6 to 0.15 mm prior to the front face of the material window. SA, synthetic apertures transmit beamformer. Error bars represent standard error, calculated from 41 lines of the B-mode images. Continuation of this figure is found in Fig. S7 in the Supplementary Material. (a) Glass. (b) COC. (c) Ibidi. (d) PMP.

Figure 8 shows the low-gain setting average dB value of the B-mode image in the region 0.6 to 0.15 mm before the material window front face as a function of the f/# of the dynamic receive focusing. Each plot corresponds to a different window material, with each curve being the acquisition conditions. The use of glass as the optical window often corresponds with the highest clutter. When varying the size of the aperture, the largest receive aperture (f/1) results in the largest clutter. This is because the aperture is wide enough to detect the off-axis intensity (see Fig. 6). Increasing the f/# (reducing the size of the detecting aperture) up to 2 or 3 decreases the effects of off-axis clutter. The clutter at larger f/#s is presumed to be from delay and sum artifacts present when imaging a specular reflector. A description of the source of delay-and-sum artifacts from specular reflectors can be found in Sec. S.4 in the Supplementary Material.

For f/2, it was found that there was a statistically significant decrease from a rayline dry acquisition on glass to all other materials acquisitions (with $p<1\times 10^{-5}$), except for the glass wet acquisitions. For the various COC acquisitions for an f/2 receive, there is a statistical significant difference between dry rayline and the two wet acquisitions (with p<0.0001) and a statistical significant difference between dry SA and wet rayline (p<0.05). However, there is no statistically significant difference between a dry SA, dry rayline, or wet SA. For Ibidi with f/2 reception, there is no statistically significant difference between any of the acquisitions. For PMP with f/2 reception, there is no statistically significant difference between dry SA, wet SA, or wet rayline.

### 3.4 Optical Point Spread Function for Different Optical Windows

**Fig. 9:**
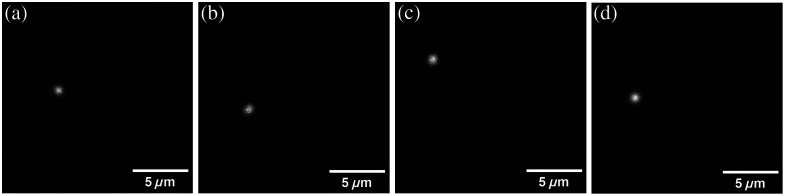
Example of the optical PSF in SHG images of a single glass bead imaged through different optical windows. (a) Glass. (b) COC. (c) Ibidi^®^. (d) PMP. Continuation of this figure is found in Fig. S8 in the Supplementary Material.

All optical window materials resulted in SHG-based optical median PSFs that were larger than the median PSF of glass. Figure 9 shows examples of the optical PSF for different materials. Table 3 lists the medians and ranges of the optical PSF for each optical window material. Only glass, COC, Ibidi^®^, PMMA, and PMP produced optical PSFs with a median below 1  μm in both x and y.

**Table 3:** Medians and ranges of the full width at half-maximum SHG images of 0.2-μm yellow-green fluorescent glass beads. COC was product code 10001999 from Microfluidic ChipShop. PMP is 125  μm thick.

Material	X-axis FWHM (median, range) (μm)	Y-axis FWHM (median, range) (μm)
Glass	(0.80, 0.45)	(0.78, 0.23)
COC	(0.92, 0.12)	(0.93, 0.13)
Ibidi^®^	(0.87, 0.12)	(0.93, 0.11)
PC	(0.97, 0.18)	(1.03, 0.41)
PET	(1.03, 0.70)	(1.06, 1.01)
PMMA	(0.93, 0.14)	(0.96, 0.31)
PMP	(0.93, 0.06)	(0.90, 0.25)

### 3.5 Effects of Optical Window Material on SHG Images of Collagen Fibers

**Fig. 10:**
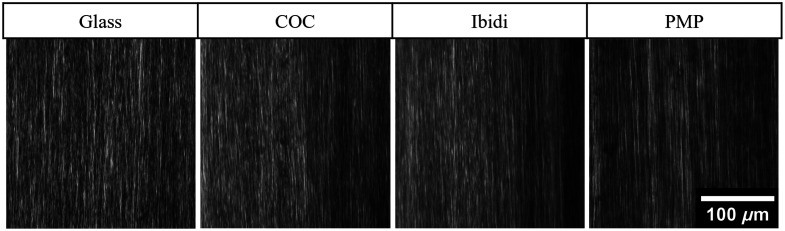
Rat tail tendon images. Each column is labeled with the material used as a coverslip. COC was 0.175 mm thick, and PMP was 0.125 mm thick. The full rat tail tendon image dataset is found in Fig. S9 in the Supplementary Material.

Figure 10 shows four example SHG images of different fields of view of rat tendon collagen fibers obtained with the different optical window materials. The top row indicates the name of the material. Table 4 gives the median and range of the measurements of collagen fiber alignment extracted from CurveAlign.^22^^,^^23^ Glass had a median alignment of 1.00, which matches the assumption that the rat tail tendon has highly aligned fibers. COC and PC also had a median alignment of 1.00. The median of the Ibidi^®^ alignment estimates was within 3% relative error of the glass alignment median estimate. The rest of the materials had median alignment measurements that had >7% error relative to glass, with the worst performer being PET, which correlates with the large PSF reported in Table 3. Indeed, the images through PET in Fig. S9 are blurry compared with the glass images.

**Table 4:** Median and range values for the automated collagen fiber alignment quantification of the four images shown in Fig. 10.

	Glass	PC	PET	PMMA	PMP	Ibidi^®^	COC
(Median, range)	(1, 0)	(1, 0.01)	(0.055, 0.04)	(0.895, 0.07)	(0.93, 0.27)	(0.965, 0.03)	(1, 0)

### 3.6 Imaging of Collagen Hydrogels

**Fig. 11:**
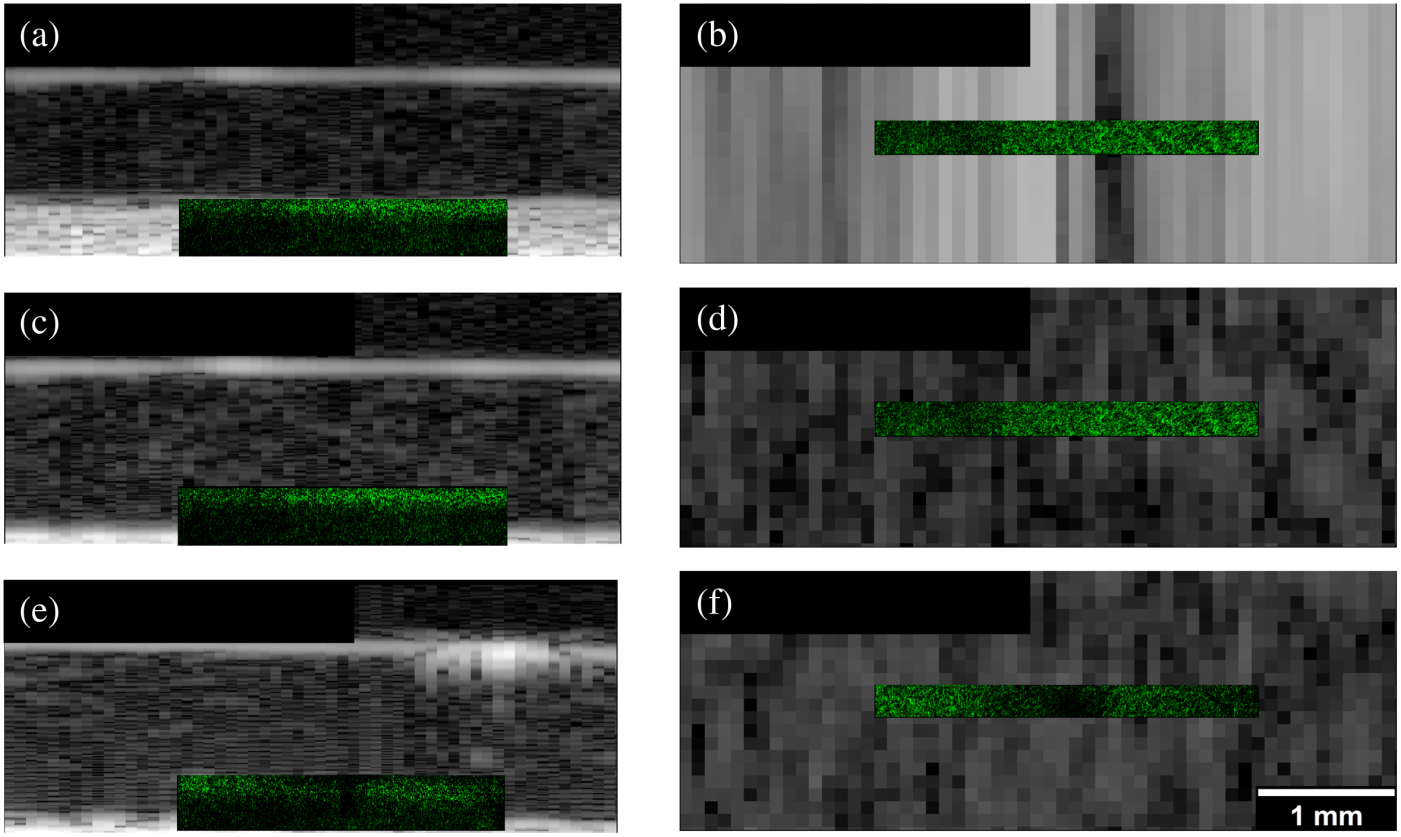
Registered slices of 3D B-mode data of a 2-mg/mL collagen hydrogel with rayline scanning and various reception beamforming f/#s. The x–z slices are located 0.4 mm above the optical window. (a) and (b) Rayline scan slices on glass with an f/1 receive. (c) and (d) Rayline scan slices on glass with an f/2 receive. (e) and (f) Rayline scan slices on COC with an f/2 receive.

Three B-mode images of the 2  mg/mL collagen hydrogel are shown in Figs. 11(a), 11(c), and 11(e). The bright line at the top of the B-mode images represents the top boundary of the collagen gels. The respective x–z and x–y slices of the 3D SHG and US data of the collagen gels are overlaid. The SHG data are registered and are to scale with the US data. The x–y slices [Figs. 11(b), 11(d), and 11(f)] of the data are from 0.4 mm above the optical window. Notice for the f/1 B-mode acquisition in Fig. 11(a), the US clutter covers practically the entire SHG imaging region. The ultrasound speckle that can be seen in Figs. 11(d) and 11(f) cannot be seen in Fig. 11(b), since Fig. 11(b) is dominated by ultrasound clutter.

### 3.7 Summary of Findings

Table 5 summarizes the findings from the acoustic and optical characterization of different combinations of optical window, transmit beamformer, and dry versus wet back-face conditions, considering the three materials COC, Ibidi^®^, and PMP. The clutter measurements in the table have been normalized by the physically focused rayline scan of a dry glass optical window (the default imaging settings used on the multimodal scope prior) and thus show the reduction of clutter in decibels when changing beamforming tactics and back face dry versus wet conditions. The largest clutter reduction from the three materials is >3.8  dB which occurs with using PMP. Unfortunately, the collagen fiber alignment error for PMP is greater than 5%, so it is not a viable optical window according to the criteria in this study. For COC and Ibidi^®^, the largest clutter reduction is 2.6 dB and occurs with physically focused dry conditions. The collagen fiber alignment error for COC and Ibidi^®^ is smaller than 5%, making these two materials good optical window candidates.

**Table 5:** Summary of values for glass and the materials with the lowest measured acoustic reflection coefficients. The average f/2 clutter measurements in the region 0.15 to 0.6 mm above the optical window are given in decibels relative to the f/2 dry rayline scan on glass.

	Glass	COC	Ibidi^®^	PMP
Reflection coefficient |Γ| (mean ± σ)	0.797 ± 0.001	0.2408 ± 0.0004	0.2560 ± 0.0005	0.0664 ± 0.0009
Physical focus dry clutter dB (mean ± σ)	0.0 ± 0.4	−2.6 ± 0.4	−2.0 ± 0.4	−3.8 ± 0.4
Synthetic focus dry clutter dB (mean ± σ)	0.2 ± 0.7	−2.3 ± 0.9	−2.0 ± 0.8	−4.2 ± 0.8
Synthetic focus wet clutter dB (mean ± σ)	−0.3 ± 0.7	−2.0 ± 0.9	−2.1 ± 0.7	−4.2 ± 0.8
SHG PSF FWHM x (median, range) (μm)	(0.80, 0.45)	(0.92, 0.12)	(0.87, 0.12)	(0.93, 0.06)
SHG PSF FWHM y (median, range) (μm)	(0.78, 0.23)	(0.93, 0.13)	(0.93, 0.11)	(0.90, 0.25)
Collagen fiber alignment (median, range)	(1, 0)	(1, 0)	(0.965, 0.03)	(0.93, 0.27)

## 4 Discussion

### 4.1 Results Overview

The goal of this work was to develop a comprehensive strategy to reduce acoustic clutter in a dual MPM/US multiscale system by minimizing optical window reflectivity and optimizing ultrasound beamforming strategies. These improvements were designed to preserve SHG image quality, particularly for quantitative collagen fiber morphology analysis. To achieve this goal, several polymer films underwent acoustic and optical testing under controlled imaging conditions. The following tests were performed on the samples: characterization of the acoustic properties, evaluation of off-axis clutter under different optical window/transmit beamforming/back-face coupling combinations, evaluation of the SHG spatial resolution by means of the point spread function, and comparison of quantitative morphological descriptions of an animal model of a collagen fiber scaffold (rat tendon). In addition, the beamforming strategies of synthetic aperture and rayline scanning were employed under high-gain settings to image a collagen hydrogel. The main findings of these tests were the following:

• PMP had the lowest reflection coefficient, followed by COC and then Ibidi^®^. Using PMP as an optical window would reduce the reflection coefficient from glass by 90%, whereas using COC or Ibidi^®^ would reduce the reflection coefficient by around 70%.
• During receive beamforming, an f/# of 2 or 3 minimizes the effects of clutter from off-axis intensity. Using COC or Ibidi^®^ reduces the average clutter by >37% (−2.0  dB) compared with the clutter produced from a glass dry rayline acquisition.
• The median of the SHG-based PSF increased when changing the optical window from glass to any other material. All materials retained a sub-micrometer PSF or were within 3% of a 1-μm FWHM median.
• From all alternative materials, COC, Ibidi^®^, and PC led to errors <5% compared with glass in the quantification of collagen alignment in SHG images of a rat tail tendon.
• Rayline scanning provides superior ultrasound imaging at high-gain settings, due to the lack of unexplained clutter compared with synthetic aperture imaging.

### 4.2 Reflection Coefficients and Effect on Acoustic Clutter

The greatest reduction in off-axis clutter measured on pre-receive beamformed RF echo signals was achieved using synthetic aperture transmit beamformers, which produce weaker, unfocused beams compared with focused ones used in rayline transmit beamforming (Fig. 6). The weaker unfocused beams reduce the acoustic force incident on the optical window, thus decreasing the ringing effect. This ringing effect was also reduced using wet conditions in combination with polymers with low acoustic reflectivity as alternative materials for the optical window. Polymers were chosen because they are known to have lower acoustic reflection coefficients in water (which depend on mass density and speed of sound) compared with glass. Table 2 contains the measured acoustic impedances of the materials. Glass was measured to have an acoustic impedance of 13.09 MRayl, which is within the range of reported acoustic impedance.^28^ The measured acoustic impedances of PMMA and PC are within 3% of reported values.^28^ The measured acoustic impedance of PET is within 6% of Mylar’s reported acoustic impedance, which are both polyester-based polymers.^29^ The PMP acoustic impedance measurements are within 8% of reported values.^30^ The acoustic impedance of COC and Ibidi^®^ is not reported in the literature. The discrepancies of the measured acoustic impedance from those in the literature could be because different grades or curing temperatures of polymers were used compared with those found in literature. Our results (Table 2) demonstrate that all investigated polymers had reflection coefficients smaller than that of glass by at least 54%, with the extreme being PMP reducing the reflection coefficient by 90%. As shown in Figs. 6(a) and 6(b), further reductions in clutter are achieved when water is added to the back face of the optical window. Except for glass, the reflection coefficient for most materials showed negligible frequency dependence. This may be due to the reflection of the back face of the glass slightly overlapping with the front face reflection, or from thin-film frequency dependency. However, the measured reflection coefficients of glass are well within the range found in the literature.

The effect of clutter on the post-receive beamformed B-mode images was quantified by averaging the B-mode image values (in dB relative to the noise floor) in a 0.45-mm-long region above the optical window. The largest values of clutter were found with large receive apertures (low receive f/#), a glass material window, and the dry imaging condition in which the opposite window face was in contact with air. A surprising finding was that a receive f/# of 2 or 3 minimized the B-mode clutter, contrary to the recommendation from a previous report that suggested a receive f/# of 9.^4^ It is hypothesized that the large receive apertures present with f/#<2 are wide enough to detect the off-axis intensity from the ringing window, as shown in Fig. 3. The smaller apertures with f/#≥2 are small enough that the off-axis intensity is not included in the image reconstruction. According to Perrot et al.,^31^ the receive f/# implemented during delay-and-sum should be selected so that the receive aperture only includes a certain number of the transducer elements in the image reconstruction. According to this strategy, the receive f/# is calculated as $f/\#=\frac{1}{2\times \tan(\alpha )}$, where α is the maximum angle of sensitivity of the elements of the aperture. For the L22-14 transducer used in this work, α=0.34  radians and $f/\#=\frac{1}{2\times \tan(0.34)}=1.41$, which is closer to the optimal clutter-reducing receive f/# of 2 and 3 found in this work. A receive f/# of 1.4 is included in Fig. 8, but this shows that this ideal f/# should not be used because it results in large acoustic clutter in the B-mode images, an issue not accounted for in the derivation of the expression for the optimal f/#.

The clutter analysis in Fig. 8 is performed with low-gain settings, preventing the highly reflective optical windows from saturating the ultrasound detecting circuitry (in particular, the amplifiers). The results in Fig. 8 suggest that, for each material, low f/#s create large clutter (>10  dB above the noise floor) in the B-mode images in the region 0.15 to 0.6 mm above the imaging window. Decreasing the f/# results in a decrease of clutter, which seems to flatten out from the f/2 to f/6 reception f/# range. It is recommended that an f/# of 2 is used to be closer to the ideal f/# while avoiding the large clutter with lower f/#s. In the low-gain settings, it was found for Ibidi that there was no statistically significant difference in the acquisition condition for an f/2 reception. For COC, there was no statistically significant difference between the dry SA and dry rayline acquisitions. It seems that a synthetic aperture acquisition and a rayline scan produce clutter reduction for COC and Ibidi that is not significantly different. There is a statistically significant decrease in clutter from the glass rayline B-mode to all other material rayline B-modes. PMP produces the lowest clutter values but does not provide accurate collagen fiber alignment quantification (see Table 5). COC and Ibidi are the two materials that provide the lowest clutter reduction from a glass-rayline acquisition while maintaining accurate collagen fiber alignment quantification.

The high-gain images of a water-filled well (Fig. 7) demonstrate the large clutter increase for an f/1 reception but also reveal that synthetic aperture derived B-mode images [Figs. 7(c) and 7(d)] have clutter that is not present in the high-gain rayline scan B-mode images [Figs. 7(e) and 7(f)] or the equivalent low-gain B-mode SA images [Figs. 8(a) and 8(b)]. From this, it is recommended that the rayline scan acquisitions be used to prevent this unexplained and unexpected clutter.

In imaging a collagen hydrogel as a model of soft tissue, it is important that the SHG imaging region overlaps the US imaging region that is not affected by clutter. Figure 11(a) shows that the acoustic clutter completely covers the SHG imaging region when a receive f/# of 1 is used. The clutter does not extend as far when f/2 receive is used, as shown in Figs. 11(c) and 11(e). The cross-sectional x–y slices of the 3D SHG and US data [Figs. 11(b), 11(d), and 11(f)] clearly show the x–y ultrasound speckle for the f/2 data, but the f/1 x–y slice is dominated by the ultrasound clutter. The f/2 receive aperture with a rayline scan does allow for a greater overlap of US and SHG imaging regions while avoiding the unexplained clutter in the SA well images [Figs. 7(c) and 7(d)]. At the high-gain settings, necessary to detect low echogenic tissue, the use of a COC or Ibidi optical window will still reduce the clutter relative to the glass optical window clutter.

Grating lobes can also contribute to the generation of off-axis clutter. Grating lobes are a spatial aliasing artifact that occurs when the element spacing is greater than the wavelength of the ultrasound wave. Grating lobes do exist natively for the L22-14v transducer, due to the wavelength of the center frequency (∼0.095  mm in water) being less than the pitch of the transducer (0.1 mm). However, the grating lobes of the center frequency occur at 71 deg from the normal direction to the transducer face. With the transducer placed 8 mm from the optical window, the arrival time of the echoes from grating lobes would be much greater than the echoes from the ringing effect of the optical window. To verify that grating lobes did not contribute to clutter in our experiments, the echo responses of the original configuration of the L22-14 transducer were simulated in Field II^32^^,^^33^ and compared with those of a “lobe-less” alternative configuration with 1/3 the pitch and 1/3 the element size of the L22-14v, but with the same transducer footprint. The pitch of the alternative configuration is 0.033 mm, which is half of the smallest wavelength in the bandwidth (1.48  mm×MHz/22  MHz∼=0.067  mm), so there are no grating lobes at any frequencies in the bandwidth. Field II simulations of various transmit and receive f/# show that there are no significant qualitative differences between the original L22-14v and the 1/3 scaled L22-14v transducers. See Sec. S.5 in the Supplementary Material for a more complete analysis of the grating lobes.

### 4.3 Optical Assessment of Alternative Optical Windows

The spatial resolution attained in the SHG scope with the different optical window materials was evaluated through the PSF. The PSF is a standard for measuring the performance of an imaging system. In the original publication by Pinkert et al.^2^ reporting the multiscale system, the PSF measured with a glass optical window was 0.81±0.14  μm in x and 0.90±0.07  μm in y, which is within the range of the values reported in Table 3. Optical windows with different materials increased the size of the PSF with respect to glass by up to 36% for PET. This loss of spatial resolution can hinder collagen fiber imaging, which requires a sub-micrometer resolution. Other than PET and PC, the median values of the material FWHM measurements provide sub-micrometer resolution, which is ideal for collagen fiber imaging.

We further analyzed the impact of the loss of spatial resolution on the quantitative analysis of collagen fiber morphology of a rat tail tendon. A rat tail tendon was utilized for its availability and previous use as a biological reference phantom.^25^ The rat tail tendon possesses highly aligned collagen fibers. Images of a rat tail tendon should appear as highly aligned in the collagen fiber quantification program CurveAlign. Overall, the use of PC, Ibidi^®^, and COC resulted in differences smaller than 5% in the analysis of collagen fiber alignment median compared with glass. Other materials resulted in errors larger than 5% compared with glass. It is important to highlight that other factors may impact the quantitative analysis of collagen fiber morphology. For example, PMP, the material with the lowest acoustic reflectivity, has a matte finish on one of the sides that did not affect the PSF measurement but aberrates the overall SHG image. This could be because the matte finish possesses a submillimeter texture that is much larger than the size of the glass bead, so the PSF was not aberrated by the matte finish. The larger SHG image of the tendon could be aberrated by the matte finish, resulting in errors in collagen fiber alignment quantification.

In summary (Table 5), COC and Ibidi^®^ with a rayline scan acquisition and f/2 reception beamforming, regardless of the wet/dry imaging conditions, showed the lowest clutter values without compromising optical image quality. Comparing the wet versus dry conditions, there was no statistically significant advantage shown for COC or Ibidi when using wet conditions. For convenience, it is recommended that dry conditions should be used. A COC or Ibidi^®^/Rayline/dry back-face condition is sufficient for COC to reduce the off-axis clutter intensity by at least 37% (−2  dB) of that obtained with glass/rayline beamforming/dry conditions.

### 4.4 Limitations

The proof-of-concept work reported here had several limitations. The list of optically clear materials tested was not exhaustive but is limited to polymers commonly used in manufacturing clear polymer lenses for optics. There may be other materials that are optically clear that could also be used as an optical window that did not satisfy the inclusion criteria in this paper. Despite this limitation, the identification of alternative optical window materials that are commercially available would facilitate access to these materials and increase their use in experimental settings based on the combination of ultrasound and optical imaging.

Regarding the acoustic characterization and ultrasound imaging findings, the reported observations are specific to the bandwidth offered by the single-element and array transducers used in these experiments. These bandwidths are relevant for high-frequency ultrasound imaging with superficial penetration. However, the experimental techniques described here could be extended to lower frequencies by accounting for the scalability to longer wavelengths at lower frequencies. It is expected that a reduction of optical window ringing using a synthetic aperture acquisition over a rayline scan will apply to lower frequency transducers because the decrease of optical window “ringing” with a synthetic aperture sequence comes from a reduction of acoustic pressure hitting the optical window. On the contrary, because the width of physically focused beams increases as frequency decreases, the focused acoustic pressure will be exerted over a wider area of the optical window, likely increasing the ringing. The reduction of delay-and-sum artifacts may not extend to lower frequencies because the axial pulse length of lower frequency transducers will become greater than the thickness of the optical window. This could mean that the reflection from the thin film at lower frequencies will be dominated by the back-face film-air interface, which has ∼100% reflectance. One might find that acoustic coupling on the back-face (i.e., the “wet” condition mentioned in Sec. 2) will become vital to reduce delay-and-sum artifacts at lower frequencies. These issues may be of relevance, for example, when using 2D matrix array transducers to obtain 3D ultrasound data, which usually operate at lower frequencies than the ones considered here. Further experimentation at lower frequencies would need to be conducted to test these hypotheses.

The clutter present in the high-gain SA reconstruction B-mode [Figs. 7(c) and 7(d)] that is not present in the high-gain rayline B-mode is currently unknown. It is especially puzzling that this clutter is not uniform across the image, but seems to vary across lateral location. One potential source could be reverberations between the optical window and the transducer. As the main acoustic medium is water, which is not as attenuating as tissue, the acoustic energy from a transmission could reverberate and be detected in future transmission sequences, which reverberations may be too low in amplitude to be detected in the low-gain settings. If this is the case, then the reverberations would be more present if the ultrasound transducer is perfectly perpendicular to the optical imaging window, and the regions of low clutter could be where the transducer is not perfectly perpendicular to the optical window, decreasing the reverberation effect. This conjecture would be difficult to test with the manual ultrasound transducer orientation methods currently used.

Regarding imaging a rat tail tendon, the described procedure is susceptible to variability due to imaging a biological sample that can shift when the coverslips are removed and replaced. Despite this variability, we were still able to identify optical window materials that provided optical imaging quality in terms of collagen fiber alignment similar to glass.

Tests of statistical significance were not performed on the differences in the PSF estimates and the automated collagen fiber alignment estimates. Due to the complexity of these experiments, only three to five data points were collected for each material in these experiments. Because of the low number of data points, statistical analysis was foregone, and the medians and ranges of the estimates were reported instead.

The direct correlation of US and MPM data will have difficulty being extended to *in vivo* applications without novel hardware development. It is feasible to indirectly correlate US and MPM *in vivo* data using landmark detection or other image registration methods. One major difference from the methods described in the study by Pinkert et al.^2^ to an *in vivo* application would be that the MPM data would be correlated with the top of the B-mode image instead of the bottom of the B-mode image. For *in vivo* 3D ultrasound data, a 2D ultrasound matrix array would be more reproducible compared with mechanically sweeping a 1D array, as is done in the original report.^2^

### 4.5 Future Work

Our group is currently investigating novel ultrasound-based techniques to track collagen fiber remodeling during several physiological and pathophysiological processes where this combined US/MPM platform will be useful. The US techniques we are using are based on the statistical and spectral (frequency-dependent) analysis of backscattered echo signals and are therefore referred to as bQUS. Several studies have demonstrated that bQUS features are sensitive to collagen fiber microstructure. For example, the backscatter coefficient, an absolute measure of echogenicity of the tissue parenchyma, has been shown to vary with the angle of the ultrasound beam with the myocytes of the myocardium.^34^ Another study investigated the use of bQUS features to track the remodeling process of the cervix during pregnancy, with the long-term goal of using these features to predict the time of parturition.^13^ The process of cervical remodeling involves drastic changes in the collagen fiber microarchitecture. Several studies in Rhesus macaques and humans have demonstrated the potential of using bQUS assessing attenuation and scattering anisotropy to monitor the changes *in vivo* and non-invasively.^12^^,^^13^^,^^35^ Thus, features of bQUS have the potential to be used as quantitative imaging biomarkers for disease detection, diagnosis, surveillance, among other uses.^36^

A quantitative imaging biomarker is a quantitative feature extracted from a medical image that is directly linked to a biological concept (i.e., collagen fiber organization) and, therefore, represents the physio- or pathophysiological status of tissue.^36^ Thus, to transform bQUS features into biomarkers for collagen fiber organization, it is key to establish the direct relationship with the specific characteristic of collagen fibers that each feature is intended to represent or describe. The improvements to the fully integrated multiscale MPM/US imaging device reported here will allow for a higher quality of directly correlated US and MPM data, enabling this multiscale system to play a primary role in the validation of bQUS features as biomarkers for collagen fiber morphology.

It is important to note that we expect that the characterization of ultrasound and optical imaging performance in the dual-modality device described in this work extends to other tissue samples beyond the ones described here. In this dual imaging modality setting, the source of clutter is the optical window due to the large variations in acoustic impedance that it introduces, not the imaging sample. Thus, the gains in clutter reduction achieved by replacing the optical window material will extend to any imaging sample. Note, however, that in conventional ultrasound imaging, clutter is generated by the object being imaged; therefore, the amount of clutter will vary with tissue sample. Regarding the optical side of the device, the measurement of the optical PSF is a general characterization of the resolution of the SHG system, and its effects will extend to other imaging samples.

The combination of optical microscopy and ultrasound imaging can be extended to other optical microscopy techniques beyond multiphoton microscopy. Although the specific image quality metrics described here, such as the PSF, are specific to SHG, the protocol that we followed could be extended to characterize the image quality of other optical microscopy techniques. Thus, the system resolution of the different optical microscopy methods would need to be investigated to ensure that the non-glass optical window will not compromise image quality.

## 5 Conclusion

This work reported an extensive acoustic and optical characterization of thin film polymers to be used as optical windows in a multiscale MPH/US imaging system. Cyclin Olefin Copolymer and the proprietary polymer produced by Ibidi^®^ are the materials that improved the ultrasound image quality while also not compromising the optical imaging modality. Acoustically, COC and Ibidi^®^ have measured reflection coefficients <0.25, which is over a 60% decrease in reflection coefficient compared with a glass optical window. An f/# of 2 or greater minimized the clutter present above the optical window. Compared with the signal intensity of a rayline scan with a glass window, COC and Ibidi^®^ achieve more than 37% (−2  dB) reduction of the average B-mode intensity directly above the optical window compared with the glass average clutter. For f/#s greater or equal to 2, there was no conclusive evidence that a reduction of clutter was present with the wet/dry conditions or the physical/synthetic transmission beamforming strategies. Indeed, there was no statistically significant difference in clutter values for the Ibidi^®^ acquisitions for and f/2 reception. Evidence from the high-gain images suggests that a rayline scan is preferable over a synthetic aperture acquisition, due to unexplained clutter in the high-gain synthetic aperture B-mode images. With respect to the change in optical window material, there is a statistically significant reduction of clutter when swapping from a glass optical window to any other window materials. Optically, COC and Ibidi^®^ provided sub-micrometer resolution and performed similarly to glass in the collagen fiber alignment quantification test of rat tail tendon images, having alignment errors <3% relative to the glass window estimates. Thus, we recommend using COC or Ibidi^®^ as optical windows for a multimodal MPM/US scope. With this window interface solution to the compromised quality of the ultrasound imaging branch of the MPM/US scope, the multimodal platform is now enabled to study the correlation of micro- and macro-structural tissue features evaluated with optical microcopy and ultrasound.

## Supplementary Material

10.1117/1.JBO.31.1.016002.s01

## Data Availability

The code and data can be found on the GitLab page: https://github.com/uw-loci/data-and-code-for-improved-interface-paper.
