# Supplementary material for: Improved imaging interfaces on a co-registered ultrasound and optical microscopy multiscale system
Source: J Biomed Opt. 2025 Dec 25;31(1):016002. doi: 10.1117/1.JBO.31.1.016002 (PMC12771023; doi:10.1117/1.JBO.31.1.016002)
Supplement: Supplementary file 1 [file JBO_031_016002_SD001.pdf]

S.1 *Acoustic characterization of thin films with a reference planar reflector*

A planar reflector with known reflection coefficient Γ will be used to provide a reference power spectrum of the pulse-echo. The reference spectrum is given by

$$S_0(k) = \left(\frac{A_0 k}{2\pi z_f}\right)^2 \left(\Gamma\, c(k) \frac{\exp\left(i2k_w(z_f)\right)}{2}\right) \iint_{-\infty}^{\infty} H(x_0, y_0) g(z_f) dx_0 dy_0 \tag{5}$$

Where A_0 is the amplitude at the center of the ultrasound beam, k is the wavenumber (with k_w being a complex wavenumber), z_f is the depth of focus of the transducer, $c(k)$ is the transfer function of the transducer, and the double integral describes the shape of the beam at the focus, as described by the theory of focused transducers[35,36]. Redefining the integral as $D(k, z_f)$, we can get the reference power spectrum by taking the magnitude squared of the reference spectrum:

$$|S_0(k)|^2 = \frac{1}{4}\left(\frac{A_0 k}{2\pi z_f}\right)^4 |\Gamma|^2 |c(k)|^2 \exp\left(-4\, \alpha_w(f) z_f\right) \left|D(k, z_f)\right|^2. \tag{6}$$

The imaginary part of the complex wavenumber k_w is interpreted as the attenuation coefficient in water once the magnitude squared is taken.

The sample front face is now placed at the focus. The sample has a different reflection coefficient Γ_s. Following the same derivation for the reference planar reflector, the power spectrum is given by

$$|S_{ff}(k)|^2 = \frac{1}{4}\left(\frac{A_0 k}{2\pi z_f}\right)^4 |\Gamma_s|^2 |c(k)|^2 \exp\left(-4\, \alpha_w(f) z_f\right) \left|D(k, z_f)\right|^2. \tag{7}$$

Taking the ratio of the power spectra given in Equation (6) and (7) at each frequency gives this relationship:

$$\frac{\left|S_{ff}(k)\right|^2}{|S_0(k)|^2} = \frac{|\Gamma_s|^2}{|\Gamma|^2}, \tag{8}$$

which can be rearranged to represent the estimator for sample reflection coefficient shown in Equation (1).

There is a fundamental relationship between the acoustic impedance of two mediums and the reflection coefficient. This relationship is

$$\Gamma_s = \frac{Z_s - Z_w}{Z_s + Z_w}, \tag{9}$$

which can be rearranged to give the estimator for acoustic impedance shown in equation (2).

The power spectrum from the back face reflection can also be derived. The ultrasound wave will transmit through the front face, reflect off the back face, and then transmit through the front face again. This results in one reflection and two transmission events. The transmission coefficient is

calculated as $|\tau|^2 = (1 - |\Gamma_s|^2)$. The wave will travel a total distance of $2 * d$ within the thin film sample, where d is the thickness of the thin film, and the attenuation of the ultrasound will appear in a term $\exp(-4\ \alpha_a(f)\ d)$. The ultrasound wave will also experience diffraction as it travels through the thin film, and the correction factor $D^*(k, z_f + d)$ will be included in the power spectrum equation. Putting these all together, the back face power spectrum can be written as

$$|S_{bf}(k)|^2 = \frac{1}{4}\left(\frac{A_0 k}{2\pi z_f}\right)^4 |\tau|^4\ |\Gamma_s|^2 |c(k)|^2 \exp(-4\ \alpha_w(f) z_f) \left|D(k, z_f)\right|^2 * \exp(-4\ \alpha_s(f) d) \left|D^*(k, z_f + d)\right|^2. \quad (10)$$

Taking the ratio of the front face power spectrum (Equation (7)) and the back face power spectrum (Equation (10)), as well as assuming that the diffraction within the thin film is negligible (i.e. $|D^*(k, z_f + d)| \cong 1$), we get the relationship

$$\frac{\left|S_{bf}(k)\right|^2}{\left|S_{ff}(k)\right|^2} = (1 - |\Gamma_s|^2)^2 \exp(-4\ \alpha_s(f) d). \quad (11)$$

This relationship can be rearranged to solve for $\alpha_s(f)$, the frequency dependent attenuation of the thin film. $\alpha_s(f)$ is in units of nepers. Multiplying both sides by 8.686 will convert to units of dB. This gives

$$\alpha_s^{dB}(f) = 8.686 \ln\left(\frac{\left|S_{bf}(f)\right|^2}{\left|S_{ff}(f)\right|^2} * \frac{1}{(1 - \Gamma_s^2)^2}\right) * \frac{1}{-4\ d}, \quad (12)$$

which can be used to estimate the linear fit of the attenuation, as shown in Equation (3). A similar derivation can be done by accounting for different reflection coefficients on each side of the film, resulting in Equation (4).

S.2 Minimizing optical scattering from Polymethyl Pentene matte-finish

Polymethyl Pentene was listed as a polymer used in optics. Thin films of PMP can be purchased from manufacturers but have a matte-finish on one side which scatters light and causes the film to be less transparent. Fluoresbrite® YG Microspheres 90.0µm were used as the test imaging subject. One slide was made where the matte-finish was facing the beads and another where the matte-finish was facing away. Figure S.1. shows the difference when the matte-finish was in contact with the mounting media vs in contact with air. We hypothesize that the improvement of clarity in the image is from better speed-of-light matching from the mounting medium and PMP matte-finish interface compared to the speed-of-light matching from the PMP matte-finish and air. The speed-of-light matching would reduce the refraction of light through the interface. If PMP were to be used as an optical window in the multimodal scope, it is recommended that the matte-finish be facing the tissue sample so the matte-finish can have better speed-of-light matching with the tissue sample, often suspended in a 2% agar hydrogel. Acoustic characterization of PMP has been done at lower frequencies (1-6MHz) using a through transmission method[37], and suggests that PMP has very promising acoustic impedance matching with water. Due to the promising acoustic properties of PMP and the fact that the optical

scattering of the matte-finish could be reduced by placing the matte-finish in contact with the imaging subject, it continued to be part of the study.

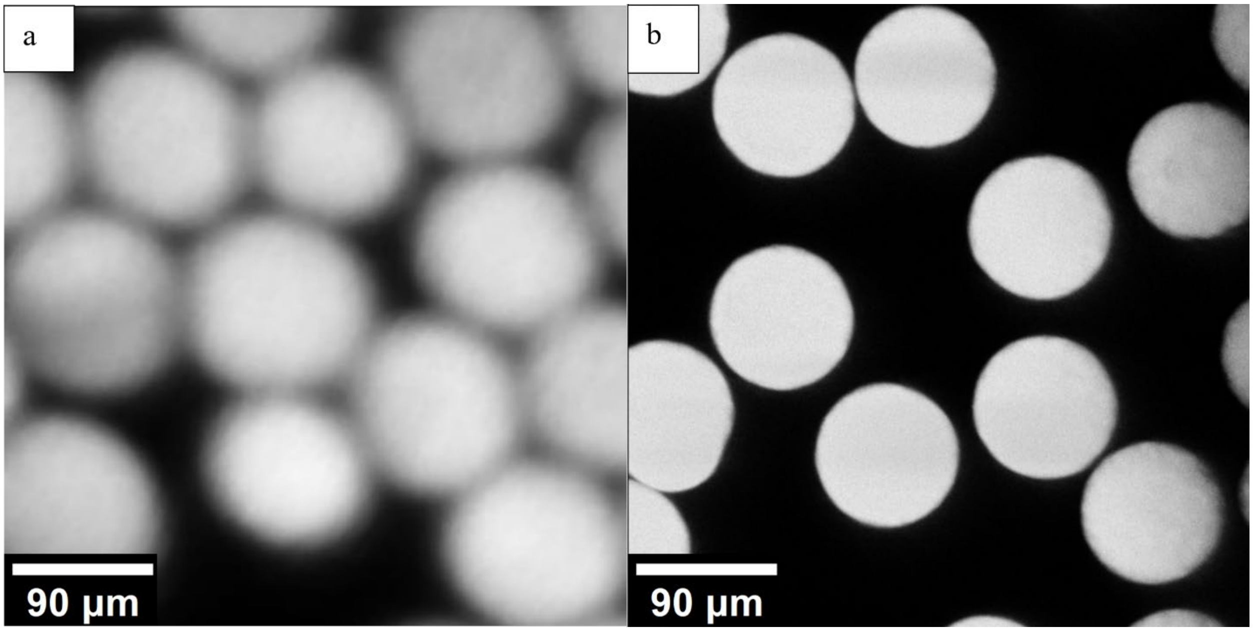

Figure S1. SHG image of 90µm fluorescent glass beads when the PMP matte finish faces away (a) and toward (b) the glass beads.

S.3 Validation of the synthetic aperture reconstruction algorithm

To check whether the B-mode synthetic aperture reconstruction is equivalent to the B-mode rayline scan, the two acquisition sequences were run on the Verasonics ultrasound simulation program. A point source was placed at the location (x,z) = (-0.05mm, 8mm), this being 8mm below the center of element 64 of the L22-14v transducer. The rayline scan uses 13 elements (an f/# of 6) for each active aperture, where time delays are applied to the elements to physically focus the ultrasound beam to a depth of 8mm, and all elements record the response of the ultrasound pulses. The synthetic aperture method pulses a single element at a time and records the response with all of the elements. The response of pulsing 13 elements with the same time delays used in the physical focusing rayline method can be reconstructed by applying time delays mathematically to the response of the pulsed elements and summing the delayed responses over

the appropriate aperture. These time delays can be performed through interpolation in the time domain or modulation in the frequency domain.

The reconstructed B-modes for the point source using a synthetic aperture reconstruction are found in Fig S8. The axial and lateral profiles of the point spread function are found in Fig. S9.

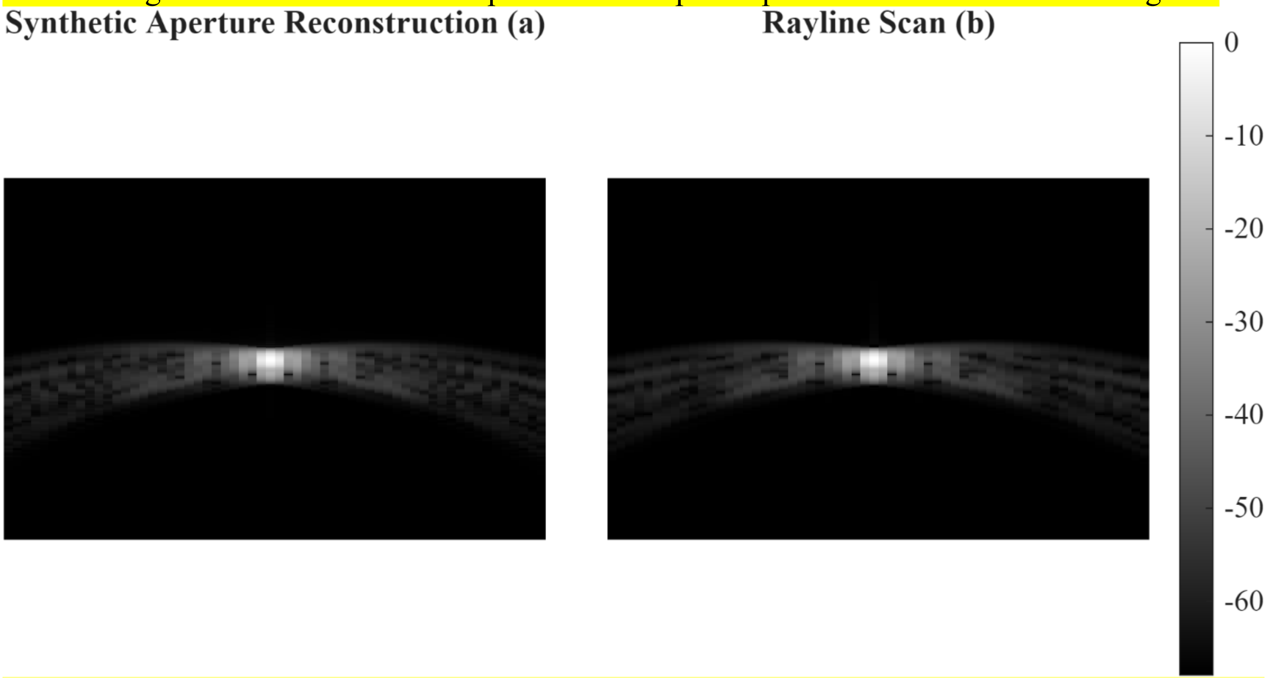

Figure S2. The B-modes of the PSF of the two acquisition algorithms, with color bar scale in dB: (a) synthetic aperture and (b) rayline.

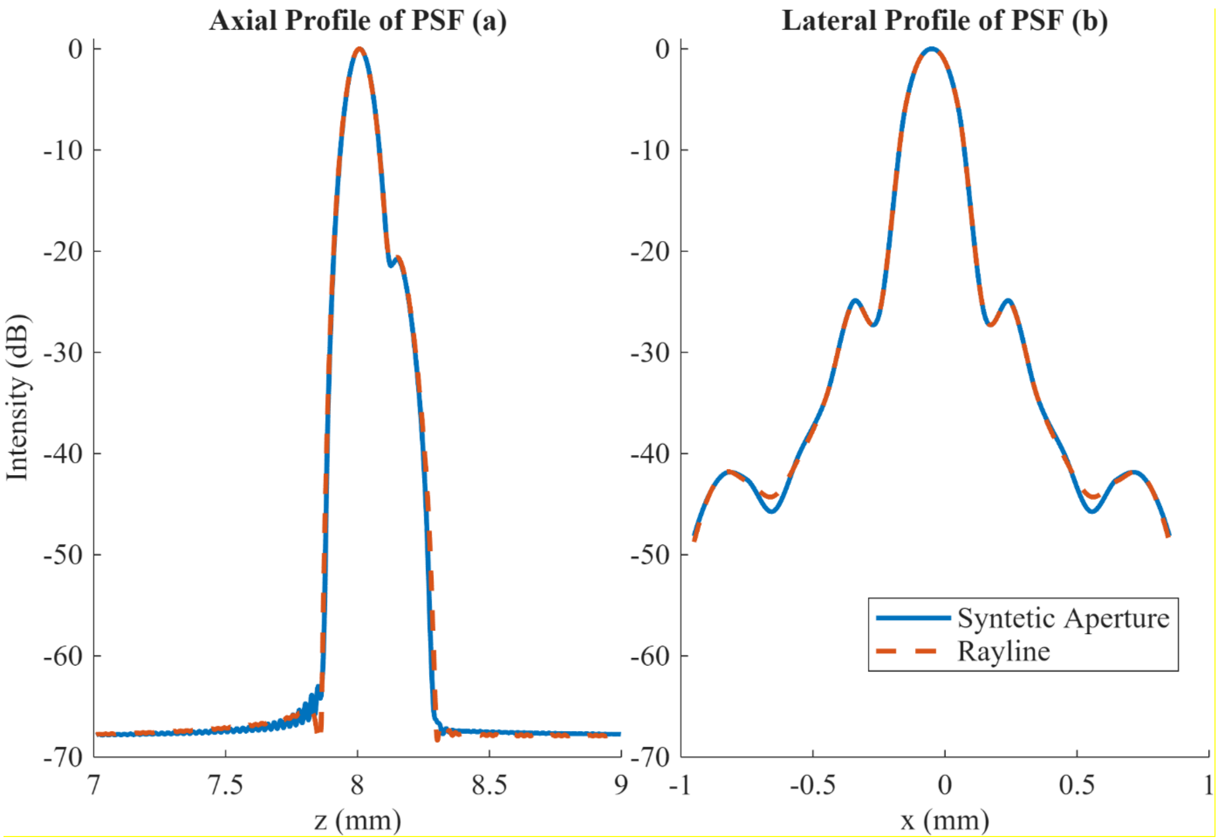

Figure S3. Profiles of the PSF of the Synthetic Aperture vs. Rayline acquisition sequences. (a) Axial profile, the A-line from element 64. (b) the lateral profile from 8mm deep.

The axial rayline full-width at half-maximum (FWHM) was measured to be 82.4µm, whereas the synthetic aperture was measured to be 82.2µm. The lateral rayline FWHM was measured to be 149.3µm, whereas the synthetic aperture lateral FWHM was measured to be 149.5µm. This means that the synthetic aperture axial and lateral PSF has less than 0.3% difference from the rayline PSF.

S.4 Delay and sum artifact from a planar reflector

DAS beamforming to create an A-line at lateral location x assumes that the only source of acoustic signal comes from scatterers located at every z depth in the reconstructed image. These scatterers are assumed to emit a spherical wave when insonified by the ultrasound pulse. Time delays are applied to 'bend' each spherical wave to be flat, meaning that summing across delayed channels will coherently sum the wavefronts from each scatterer positioned along the z-axis. Placing a planar reflector at the focus, however, reflects the wavefront that was sent from the transmission. This often means that the wavefront coming from the focus depth is not fully a spherical wave, and the time delays that would flatten a spherical wave emanating from (x,z) don't properly flatten the reflected wavefront. Figure S1 shows RF frames from Field II simulations of a f/6 transmission focused at 8mm and centered at the center of the transducer. The wavefront from a point scatterer (Fig. S1.(c)) curves downward more than the reflected

wavefront from a planar reflector (Fig. S1.(a)). When the time delays are applied, the spherical wave emanating from the point scatterer is flattened (Fig. S1.(d)), but the reflected wavefront is now bent upwards (Fig. S1.(b)). When summed across channels, this upward bent wavefront will contribute signal to the region prior to the planar reflector. This effect will be minimized when using a small receive aperture (Fig. S2, f/6 receive apertures). When using larger receive apertures, more upward bent signal will be summed into the A-line, resulting in a rising noise floor (Fig. S2, f/1 receive apertures).

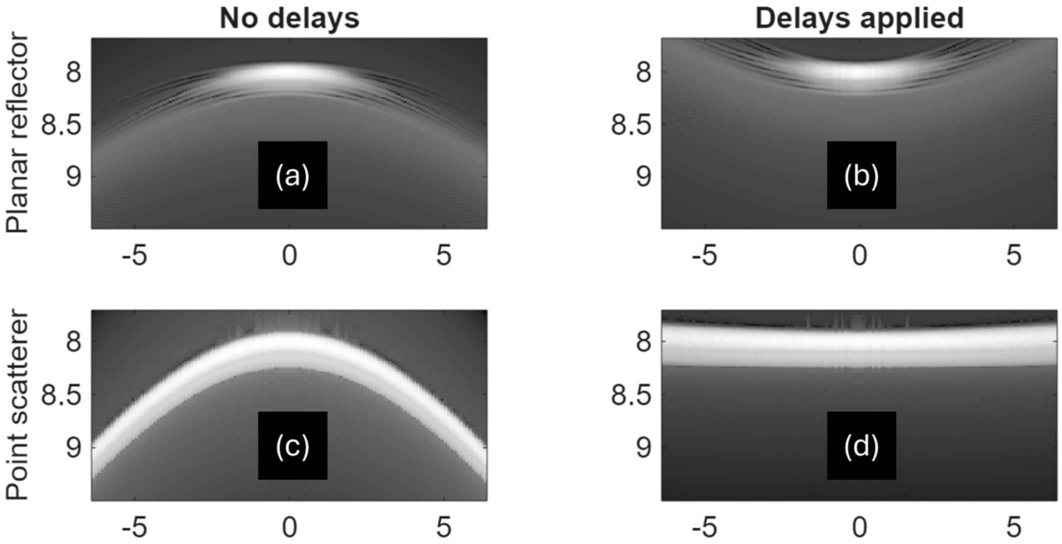

Figure S4. Field II simulations of a f/6 transmission focused a 8mm depth, and centered at 0mm. Axes are in mm. (a) RF frame from Reflected wavefront from a planar reflector placed at 8mm. (b) Delays and sum delays applied to (a). (c) RF frame from a point scatterer placed at (0,8), emitting a spherical wave. (d) delays applied to (c).

S.5 Field II Grating Lobe Simulations

As mentioned in the methods section, a plane of scatterers at 8mm depth was imaged with a simulated L22-14 transducer, and a modified L22-14 transducer with 1/3 element width and 1/3 element pitch with 388 elements. The modified L22-14 transducer will have no grating lobes at any of the frequencies in the bandwidth.

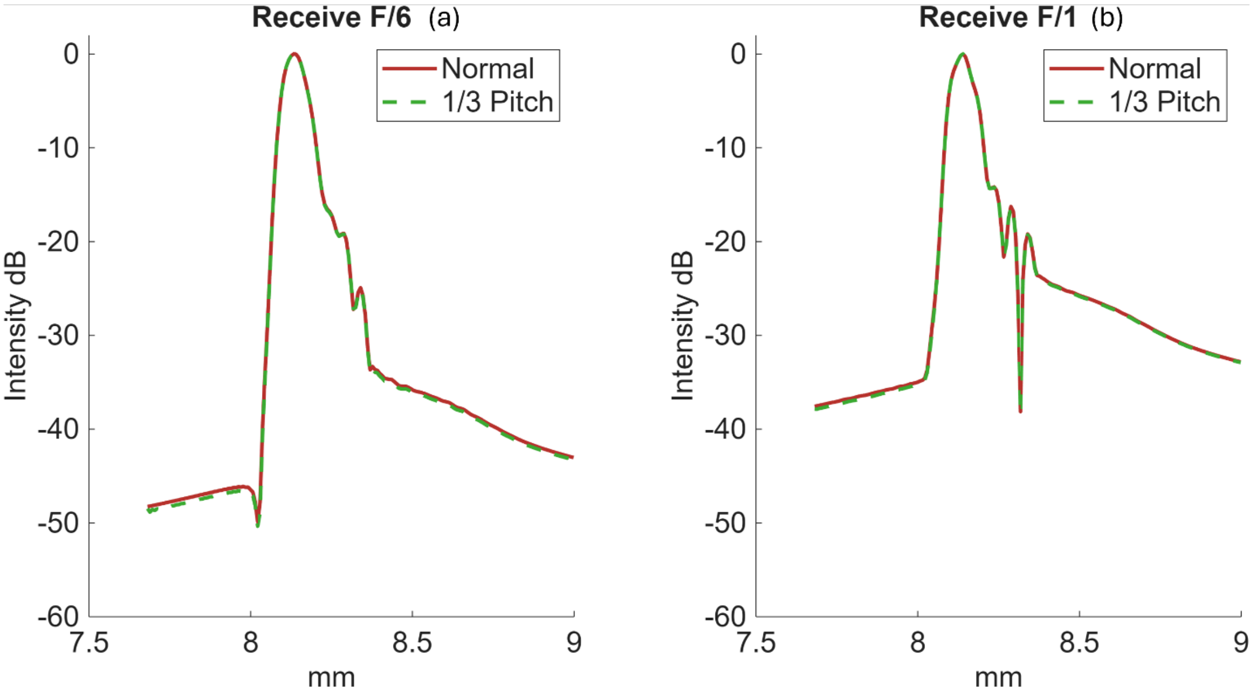

Figure S5. F/6 transmission. Field II average a-lines comparing the 128 element L22-14 (normal) with the 388 element L22-14 (1/3 pitch). (a) F/6 reception. (b) F/1 reception.

Originally, it was thought that the acoustic clutter prior to the optical window was due to off-axis clutter from grating lobes. Grating lobes do exist natively for the L22-14v transducer, due to the wavelength of the center frequency (~0.095 mm in water) being less than the pitch of the transducer (0.1mm). However, the grating lobes of the center frequency are at 71 degrees; with the transducer placed 8mm from the optical window, echoes produced by the grating lobes do not arrive to the transducer much later than the echoes from the optical window. To verify that grating lobes are not an issue, a transducer was designed in Field II with 1/3 the pitch and 1/3 the element size of the L22-14v, but with the same transducer footprint. The pitch of this 1/3 scaled transducer is 0.033 mm, which is half of the smallest wavelength in the bandwidth {1.48mm*MHz^-1 /(22 MHz) ~= 0.067mm}, so there are no grating lobes at any frequencies in the bandwidth. Field II simulations of various transmit and receive f/# show that there is no significant qualitative differences between the original L22-14v and the 1/3 scaled L22-14v transducers (see figure 8), meaning that the haze/clutter reported in figure 1 is not due to grating lobes. However, the rising signal in the region prior to the plane of scatterers (placed at 8mm depth) does highlight the delay-and-sum (DAS) artifacts due to imaging a planar reflector.

S.6 Continuation of Figures

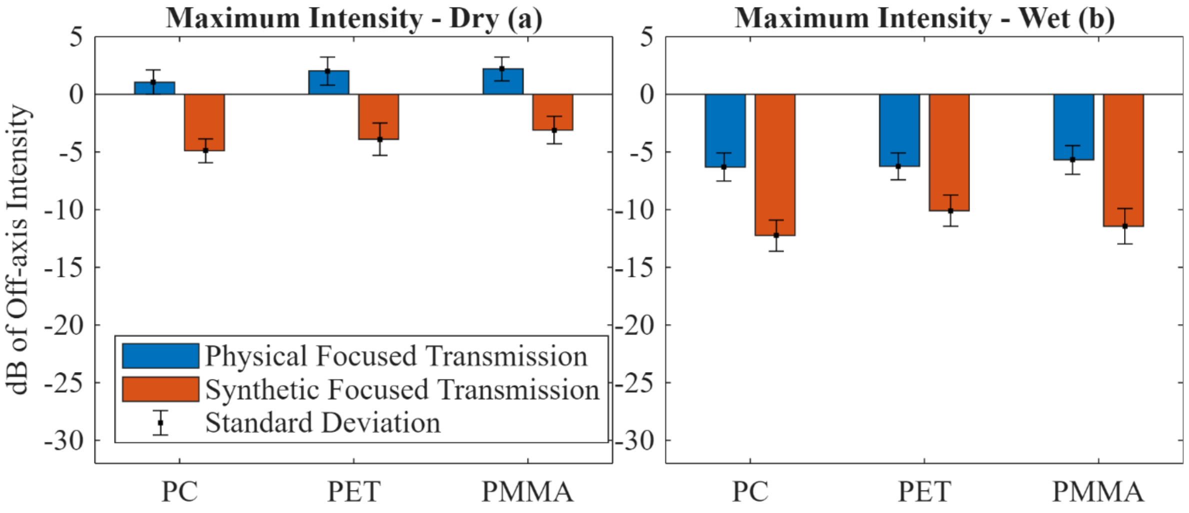

Figure S6. Continuation of Fig. 6. Average intensity from acoustic clutter produced by different material windows and beamforming approaches (physically vs. synthetically focused). (a) Dry conditions (SHG-side face of the window on air) and (b) Wet conditions (SHG-side face of the window in water).

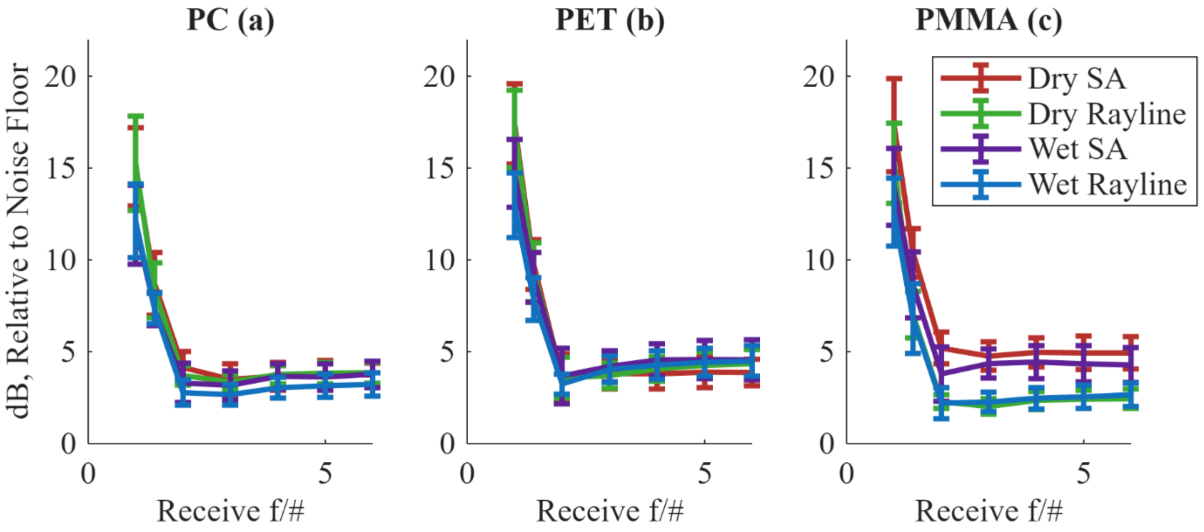

Figure S7. Continuation of Fig. 8. Mean values for the average intensity of signal in the region 0.6 to 0.15mm prior to the front face of the material window. SA: Synthetic aperture transmit beamformer. Error bars represent standard error, calculated from 41 lines of the B-mode images.

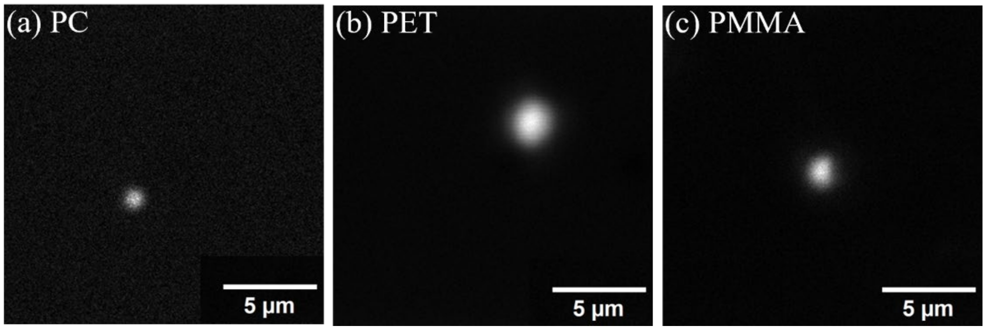

Figure S8. Continuation of Fig. 9. Example of the optical PSF in SHG images of a single glass bead imaged through different optical windows: (a) PC, (b) PET, (c) PMMA.

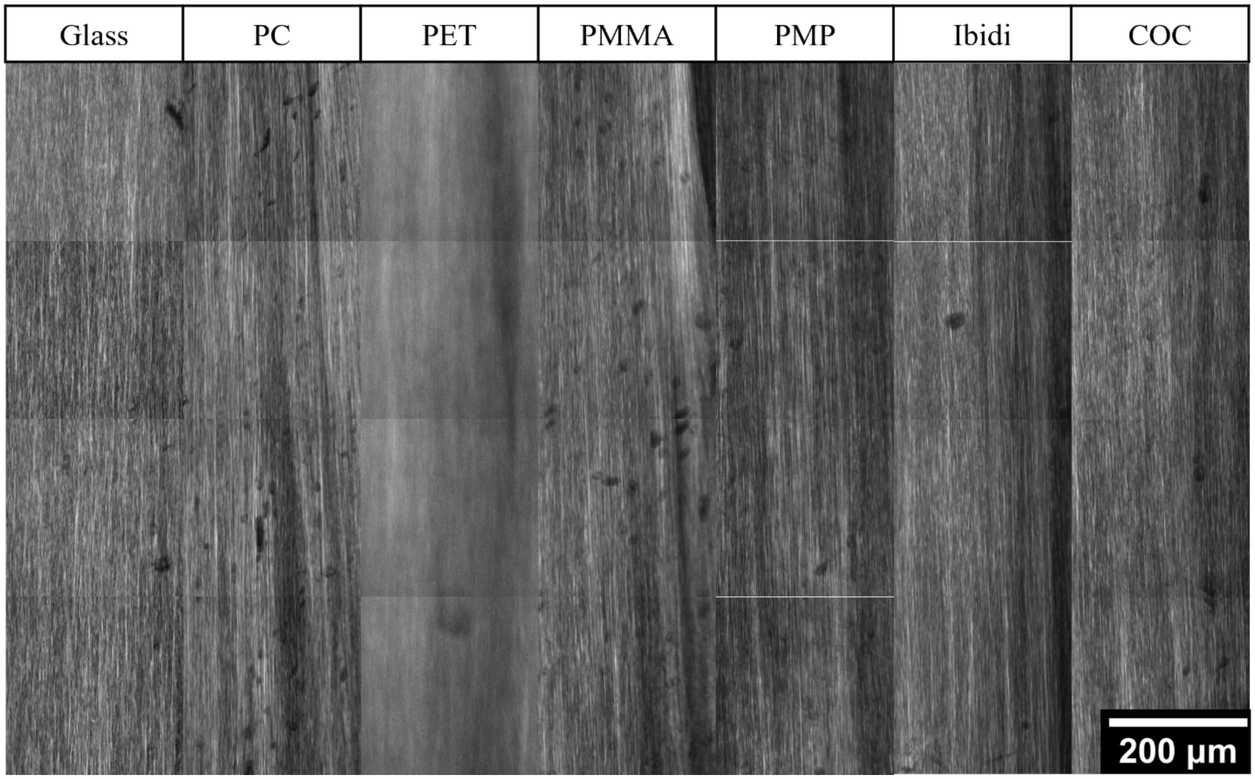

Figure S9. Continuation of Fig. 10. Rat tail tendon images. Each column is labeled with the material used as a coverslip. COC was 0.175mm thick, PMP was 0.125mm thick.
